# Mitophagy and Immune Infiltration in Primary Sjögren’s Disease: Insights from Bioinformatics Analysis

**DOI:** 10.3390/ijms27083365

**Published:** 2026-04-09

**Authors:** Liqiong Hou, Gaxue Jiang, Yanfei Chen

**Affiliations:** 1 Department of Rheumatology, First Hospital of Lanzhou University, Lanzhou 730000, China; chenyanfei@163.com; 2 Heart Center, First Hospital of Lanzhou University, Lanzhou 730000, China; 15095359123@163.com

**Keywords:** primary Sjögren’s disease, biomarkers, bioinformatics analysis, immune infiltration, LASSO, random forest, mitophagy

## Abstract

Primary Sjögren’s disease (SjD) is characterized by lymphocyte infiltration into exocrine glands. Mitochondrial dysfunction is a critical pathological mechanism underlying SjD, and mitophagy plays a vital role in clearing damaged mitochondria. This study used bioinformatic analysis to explore the potential roles of mitophagy-related genes in SjD pathogenesis and immune infiltration. Bioinformatic analysis was performed on the SjD microarray datasets to identify differentially expressed genes (DEGs). Mitophagy-related DEGs were selected and analyzed using functional enrichment, protein–protein interaction (PPI) networks, and machine learning (Least Absolute Shrinkage and Selection Operator [LASSO] and Random Forest) to identify hub genes. Their diagnostic value was assessed by receiver operating characteristic (ROC) curves. Immune infiltration and its correlation with hub genes were also evaluated. Hub gene expression in the salivary glands of patients was validated using qRT-PCR. Regulatory networks were also predicted. Three hub genes (*GABARAPL1*, *PINK1*, and *SQSTM1*) were identified. They showed high diagnostic specificity and were downregulated in SjD salivary glands. Immune infiltration analysis revealed increased levels of activated natural killer (NK) cells, memory B cells, plasma cells, CD8+ T cells, Tfh cells, and M1 macrophages, but decreased levels of Tregs and M2 macrophages. Hub gene expression was correlated with specific immune cell subsets. Regulatory network predictions highlighted potential upstream regulators and therapeutic compounds. This study identified three mitophagy-related hub genes linked to immune dysregulation in SjD, providing novel insights into disease mechanisms and potential therapeutic targets.

## 1. Introduction

Primary Sjögren’s disease (SjD) is a chronic autoimmune disease characterized by lymphocytic infiltration of exocrine glands, leading to their dysfunction. The clinical manifestations include dry mouth and eyes, fatigue, and joint pain. SjD not only affects exocrine glands but can also damage multiple organs, including the joints, liver, kidneys, and lungs, ultimately resulting in irreversible tissue damage. Studies have shown that the risk of lymphoma in patients with SjD is 5–10% higher than that in healthy individuals, and the incidence of other tumors is also significantly increased, posing a serious threat to patient health [1,2,3]. Currently, SjD is treated using empirical symptomatic therapy; however, no disease-modifying antirheumatic drugs (DMARDs) are available to target disease progression, highlighting the urgent need for innovative treatment approaches.

Mitophagy is a crucial process by which cells selectively remove damaged mitochondria, and its key role in various diseases has gained increasing attention in recent years. Research has indicated that changes in mitochondrial structure and function are significant triggers of inflammation [4]. In the salivary glands of patients with SjD, mitochondrial swelling and dysfunction are significantly correlated with disease severity [5]. Additionally, abnormalities in CD4+ T-cell differentiation and function in autoimmune diseases (such as systemic lupus erythematosus) are closely related to defects in mitophagy [6,7,8]. However, no studies have systematically explored the relationship between abnormal lymphocyte activation and mitophagy in patients with SjD.

This study aimed to analyze the transcriptomic profiles of mitophagy-related genes in SjD to reveal the correlations among mitophagy, SjD, and immune infiltration. This research provides new insights into the pathogenesis and intervention strategies for SjD. We used the Gene Expression Omnibus (GEO) database, specifically the GSE23117 and GSE40611 datasets, to create a new microarray dataset to identify differentially expressed genes (DEGs) in SjD. Subsequently, we conducted an intersection analysis of these DEGs with mitophagy-related genes to identify mitophagy-associated DEGs. We used machine learning methods to screen for hub genes related to mitophagy in SjD, followed by functional and pathway enrichment analyses using Gene Ontology (GO), Kyoto Encyclopedia of Genes and Genomes (KEGG), and protein–protein interaction (PPI) network analyses. Furthermore, we validated the expression levels of mitophagy-related genes in patients with SjD compared to those in healthy individuals and investigated the relationships between these genes and immune infiltration to gain a deeper understanding of the immune processes in SjD. Finally, we constructed transcription factor (TF)-microRNA (miRNA) regulatory networks and protein-compound networks for hub genes to support future research on the regulatory mechanisms of these genes.

## 2. Results

### 2.1. Identification of Differentially Expressed Genes Linked to Mitochondrial and Immune Dysfunction in Sjögren’s Disease

To identify DEGs in the salivary glands of patients with SjD, raw gene expression data were retrieved from the GEO database, focusing on the GSE23117 and GSE40611 datasets. After data preprocessing and cleaning, batch effect correction was applied (Figure S1A,B), and the expression matrices from both datasets were normalized. The boxplots suggest near-linear trends in the processed data (Figure S1C,D). Using thresholds of *p* < 0.05 and |logFC| > 1, DEGs were extracted from the integrated dataset. This analysis identified 942 DEGs, including 474 upregulated and 468 downregulated genes (Figure S1E,F).

To investigate the biological functions of the DEGs, GO and KEGG enrichment analyses were conducted with the R package “cluster Profiler.” (version 4.17.0).The most significantly enriched GO terms were extracellular leakage, unsaturated fatty acid biosynthesis, arachidonic acid metabolism, leukocyte-endothelial adhesion, and macrophage-derived foam cell differentiation. These processes are closely related to energy metabolism, particularly mitochondrial function, extracellular leakage, morphogenesis, and leukocyte adhesion. Mitophagy is essential for removing damaged mitochondria and maintaining cellular homeostasis.

**Figure 1:**
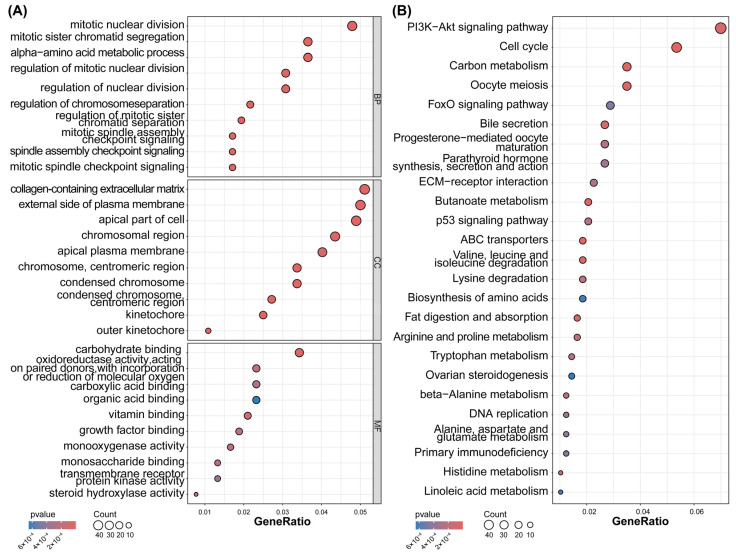
Functional enrichment analysis of 942 DEGs in salivary glands from patients with Sjögren’s disease and the control group. (**A**) GO and (**B**) KEGG pathway enrichment results. Each bubble represents a significantly enriched term (FDR-adjusted *p*-value < 0.05). The bubble size corresponds to the Count of DEGs in the term, the color gradient indicates the −log_10_(*p*-value), and the *y*-axis represents the GeneRatio (defined in Section 4).

The molecular function (MF) terms from the GO analysis predominantly encompassed energy metabolism, signaling pathways, and autophagy regulation. Fatty acid transport and the AMPK/mTOR pathway are pivotal in mitophagy and cellular homeostasis. KEGG pathway analysis further linked the DEGs to energy metabolism, stress responses, immune activity, and cell death, with notable enrichment in the PI3K-Akt and p53 signaling pathways. These pathways reflect immune metabolic disturbances in SjD and highlight the potential involvement of dysregulated mitophagy in exacerbating immune dysfunction and its clinical manifestations (Figure 1).

### 2.2. Identification of Five Mitophagy-Related DEGs and Their Enrichment in Autophagic Pathways

**Figure 2:**
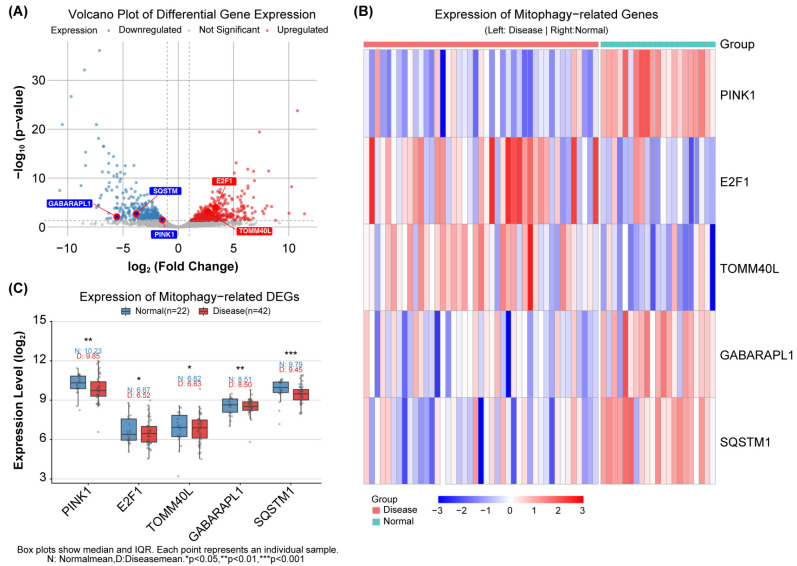
Analysis of mitophagy-related 5 DEGs between primary Sjögren’s disease and healthy control groups using integrated datasets GSE23117 and GSE40611. (**A**) Volcano plot of mitophagy-related DEGs from the integrated microarray datasets. Red and blue points represent significantly upregulated and downregulated genes, respectively (screening criteria: |log_2_FC| > 1.0 and FDR-adjusted *p*-value < 0.05). Key mitophagy-related DEGs are labeled. (**B**) Heat map showing the expression patterns of the 5 identified mitophagy-related DEGs across all samples. Rows: genes; columns: samples (left: pSS; right: normal). Color scale indicates Z-score normalized expression (blue: low; red: high). (**C**) Box plots comparing the expression levels of the 5 mitophagy-related DEGs between primary Sjögren’s disease and healthy control groups, based on microarray data. * *p* < 0.05, ** *p* < 0.01 and *** *p* < 0.001 vs. Normal group.

To identify the mitophagy-related genes differentially expressed in the salivary glands of patients with SjD, an intersection analysis was performed between the previously identified DEGs and 104 mitophagy-related genes from the KEGG pathway hsa04137. Five mitophagy-like DEGs were identified, comprising two upregulated and three downregulated genes (Figure S2A). The expression patterns of these genes are shown in a volcano plot (Figure 2A) and a heatmap (Figure 2B), based on the microarray data from GSE23117 and GSE40611. Figure 2C compares the expression levels in SjD and control salivary gland tissues. The upregulated genes were *E2F1* and *TOMM40L*, whereas *PINK1*, *SQSTM1*, and *GABARAPL1* were downregulated (Figure 2C).

GO and KEGG enrichment analyses were performed to explore the functional roles of the five mitophagy-related DEGs. GO analysis revealed that these genes were predominantly associated with macroautophagy and mitophagy (biological process, BP); mitochondrial outer membrane and its translocase complex (cellular component, CC); ubiquitin modification; transcriptional regulation; and neurotransmitter receptor binding MF (Figure S3A). KEGG enrichment analysis indicated strong links between mitophagy, neurodegenerative disorders, and cellular senescence (Figure S3B). A circular diagram summarizes the top five KEGG pathways enriched in the five mitophagy-linked DEGs (Figure S3C).

Next, a PPI network was generated for the five mitophagy-related DEGs using the STRING and Gene MANIA databases to illustrate the functional connections among these genes (Figure S4A,B).

### 2.3. Selection of Hub Genes and Diagnostic Value

**Figure 3:**
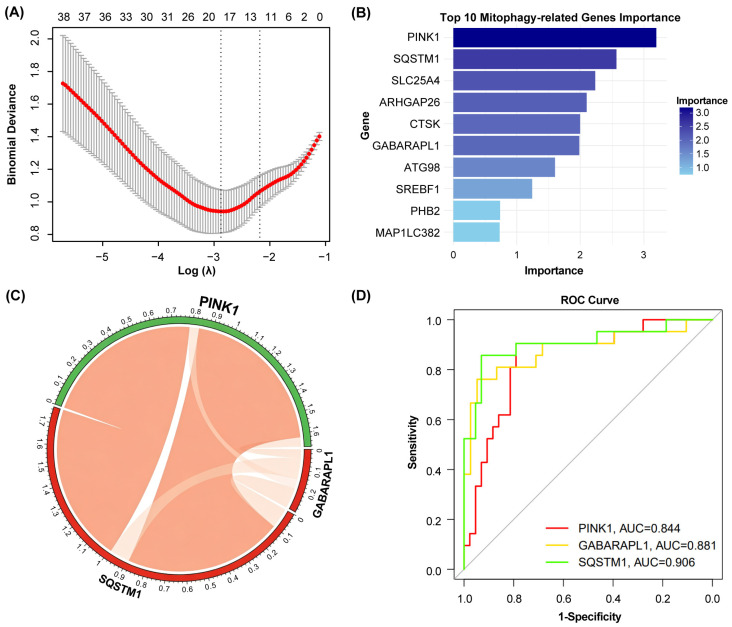
Machine learning-driven identification and diagnostic validation of mitophagy-related hub genes in primary Sjögren’s disease. (**A**) Feature selection using LASSO logistic regression. The analysis was performed using the R package “glmnet” (version 4.1.10). The optimal penalty parameter (λ) was determined via 10-fold cross-validation. The left dashed vertical line indicates the λ value that gives the minimum cross-validation error (λ.min). The right dashed vertical line indicates the largest λ value within one standard error of the minimum error (λ.1se), which selects a more parsimonious model. (**B**) Ranking of the top 10 genes by the Random Forest algorithm based on feature importance. Gene importance was quantified as the mean decrease in the Gini index (MDGI) using the R package “randomForest” (version 4.7.1.2). A higher MDGI value indicates a greater contribution of the gene to classifying Sjögren’s disease samples versus controls. The top 10 genes were selected for further intersection analysis. (**C**) PPI network of the three identified hub genes. Interactions were assessed using the STRING database. The network was visualized with a circular layout. (**D**) Diagnostic performance evaluation of individual hub genes. ROC curves were generated using the “pROC” package (version 1.18.5)in the independent validation cohort (GSE173808). The area under the curve (AUC) for each gene is shown. An AUC > 0.6 was considered indicative of diagnostic significance. Statistical significance was defined as a two-sided *p* < 0.05.

We used Least Absolute Shrinkage and Selection Operator (LASSO) regression and random forest approaches to screen for mitophagy-associated hub genes in SjD. LASSO regression selected 12 candidate genes (Figure 3A), and the random forest algorithm ranked the top 10 genes (Figure 3B). The intersection of the two gene sets yielded three overlapping hub genes: *SQSTM1*, *GABARAPL1*, and *PINK1* (Figure S2B).

To assess the diagnostic potential of these hub genes, receiver operating characteristic (ROC) analysis was conducted using the “pROC” package (version 1.18.5) on the GSE173808 cohort. The area under the curve (AUC) values were 0.906 for *SQSTM1*, 0.881 for *GABARAPL1*, and 0.844 for *PINK1* (Figure 3D), indicating high specificity of all three mitophagy-related genes for SjD.

### 2.4. Validation of Hub Gene Downregulation and Its Correlation with an Altered Immune Microenvironment

**Figure 4:**
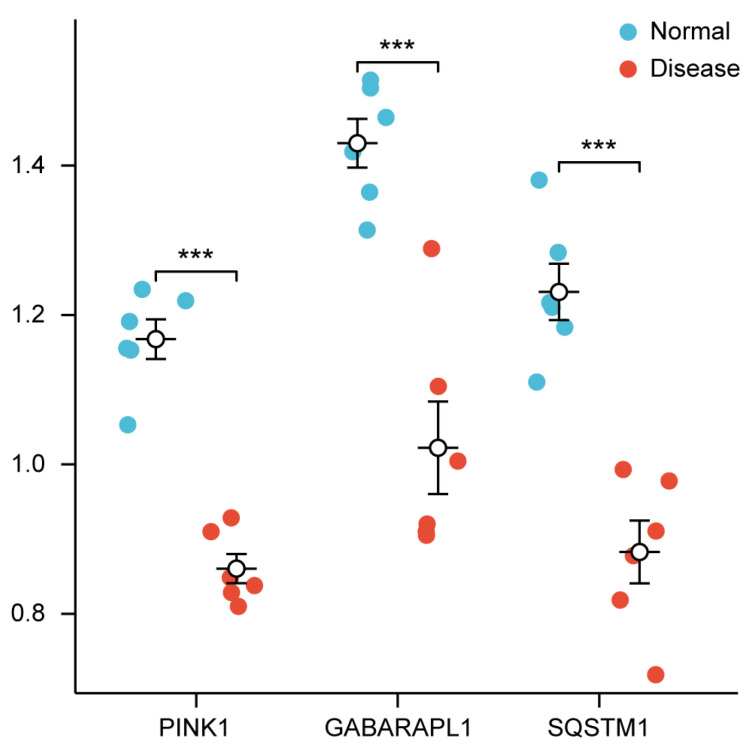
mRNA expression levels of three mitophagy-related hub genes in salivary gland tissues from primary Sjögren’s disease patients and healthy controls. mRNA expression levels of *PINK1*, *GABARAPL1*, and *SQSTM1* in salivary gland tissues from Primary Sjogren’s disease (*n* = 6) and healthy controls (*n* = 6). Each dot represents an individual sample, and horizontal lines indicate means ± SEM. Statistical significance was determined by two-tailed Student’s *t*-test; *** *p* < 0.001 vs. healthy controls.

To validate the bioinformatics results, we measured the mRNA expression levels of three mitophagy-related hub genes (*GABARAPL1*, *PINK1*, and *SQSTM1*) in salivary gland tissues from patients with primary SjD and healthy controls. Consistent with the computational predictions, all three genes were significantly downregulated in SjD tissues (Figure 4).

**Figure 5:**
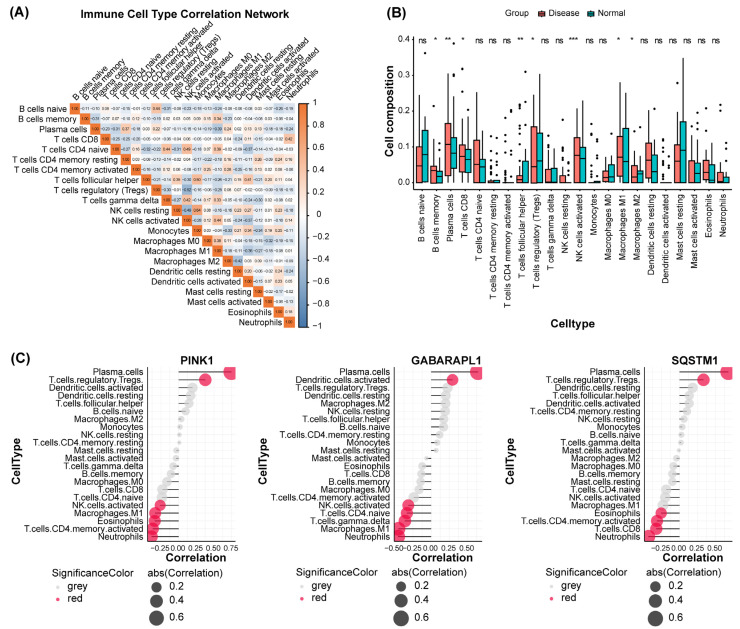
Immune cell infiltration landscape and its correlation with mitophagy-related hub genes in primary Sjögren’s disease. (**A**) Correlation network of immune cell infiltration in primary Sjögren’s disease salivary gland tissues. Pairwise Spearman correlations between 22 immune cell subsets were calculated using ssGSEA-derived infiltration scores. Color intensity indicates correlation strength (blue: negative; orange: positive); absolute values ≥ 0.2 are shown. (**B**) Abundance of immune cell infiltration in primary Sjögren’s disease patients vs. healthy controls. Immune cell proportions were quantified by ssGSEA. Data are presented as means ± SEM for each group. Statistical significance was assessed by two-tailed Student’s *t*-test; ns (not significant), * *p* < 0.05, ** *p* < 0.01, *** *p* < 0.001. (**C**) Correlation between mitophagy-related hub genes and immune cell infiltration. Significant correlations (*p* < 0.05) between *PINK1*, *GABARAPL1*, *SQSTM1* and 22 immune cell subsets are shown. Correlation coefficients (Spearman’s rho) are displayed as circles; size reflects absolute correlation magnitude (0.2, 0.4, 0.6), and color indicates direction (red: positive; grey: not significant).

Given the autoimmune pathogenesis of SjD, we performed immune infiltration analysis using the GSVA package (version 2.4.1). In this study, we evaluated 22 immune cell subtypes. The correlation matrix of the abundance levels is shown in Figure 5A. Compared to healthy controls, SjD tissues exhibited significantly higher infiltration of activated NK cells, memory B cells, plasma cells, CD8 + T cells, follicular helper T cells, and M1 macrophages, whereas regulatory T cells (Tregs) and M2 macrophages were markedly reduced (Figure 5B).

We further explored the correlation between hub gene expression and immune cell infiltration. The expression levels of *PINK1*, *GABARAPL1*, and *SQSTM1* were positively correlated with plasma cells and regulatory T cells (Tregs). In contrast, *PINK1* expression was negatively correlated with the number of activated CD4 + T cells, NK cells, and M1 macrophages. *GABARAPL1* was inversely correlated with activated NK cells and M1 macrophages, whereas *SQSTM1* was negatively correlated with activated CD4 + and CD8 + T cells (Figure 5C). All these immune cell types have established roles in the pathogenesis of SjD. Taken together, these findings indicate a link between the expression of mitophagy-related hub genes and a dysregulated immune microenvironment in SjD.

### 2.5. Association of Hub Genes with Mitochondrial Energy Metabolism Pathways

**Figure 6:**
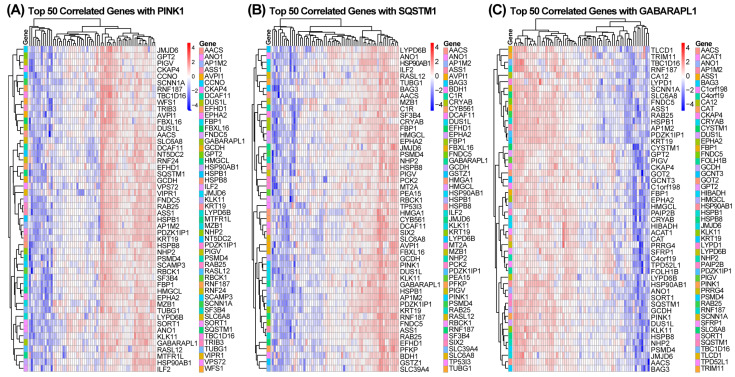
Heatmap showing the top 50 genes positively correlating with three mitophagy-related hub genes. (**A**) Heatmap of 50 genes positively correlating with *PINK1*. (**B**) Heatmap of 50 genes positively correlating with *SQSTM1*. (**C**) Heatmap of 50 genes positively correlating with *GABARAPL1*. Color scale: red = high, blue = low.

**Figure 7:**
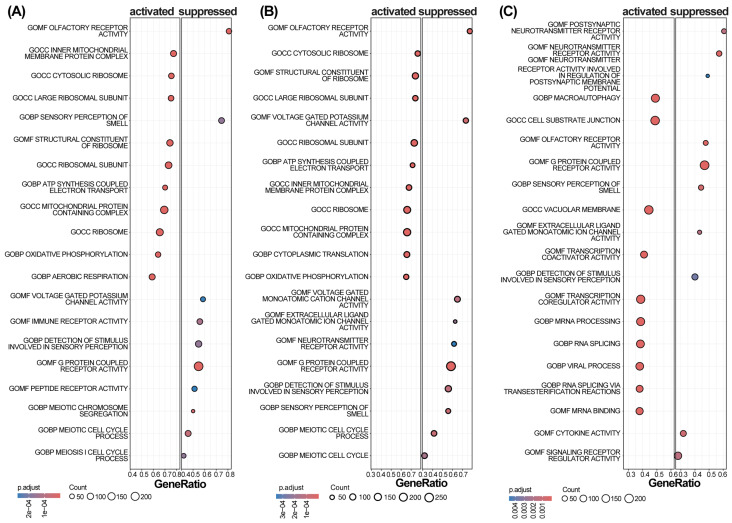
GSEA identifies key signaling pathways associated with *PINK1*, *SQSTM1*, and *GABARAPL1* in primary Sjögren’s disease. (**A**–**C**) Top 20 enriched GO terms for each hub gene. Dot size = gene count, color = p.adjust, position = activated (left) or suppressed (right). Only pathways with p.adjust < 0.05 are shown.

We performed a correlation analysis between the three hub genes and all other expressed genes. To identify and visualize the co-regulated gene networks most strongly associated with each hub gene, the top 50 positively correlated genes are presented in a heat map (Figure 6). Based on these correlation results, single-gene set enrichment analysis (GSEA) was subsequently performed to identify the signaling pathways linked to each hub gene. Figure 7 summarizes the 20 main pathways enriched for different hub genes. These results suggested that all three hub genes were closely associated with mitochondrial energy metabolism. Therefore, it is plausible that these genes may influence cell cycle and cell death signaling in SjD by modulating energy-metabolism-driven mitophagy.

### 2.6. Prediction of Upstream Regulators and Therapeutic Compounds for the Hub Genes

To identify potential upstream regulators, the Reg Network database was used to predict the miRNAs and transcription factors that target hub genes (Figure S5). In addition, compounds associated with hub genes were predicted as therapeutic candidates for SjD (Figure S6). A total of 202 compounds were obtained, and both the compounds and their corresponding target genes were ranked according to degree and betweenness centrality (Supplementary Table S2).

## 3. Discussion

SjD is an autoimmune disorder characterized by lymphocytic infiltration of exocrine glands. Its pathogenesis is closely linked to cellular metabolic dysregulation, in which mitochondria play a central role. In patients with SjD, elevated levels of oxidative stress biomarkers and pro-inflammatory cytokines have been observed [9], along with impaired autophagy [10] and autophagic alterations in the salivary glands [11].

When autophagy and mitophagy are compromised, damaged mitochondria accumulate, leading to excessive ROS production that contributes to inflammation [12]. These dysfunctional mitochondria release damage-associated molecular patterns (DAMPs), such as mitochondrial DNA (mtDNA), which activate inflammatory pathways via pattern recognition receptors, including the NLRP3 inflammasome, TLR9, cGAS/STING, and ZBP1 [13]. Notably, oxidative stress can also trigger NLRP3 inflammasome activation [14], and mitochondrial contents are highly immunogenic [15].

The critical role of mitochondrial integrity in SjD was underscored by Li et al. [5], who linked mitochondrial dysfunction to the immune microenvironment in salivary glands of primary Sjögren’s disease patients. They documented altered mitochondria-related gene expression, downregulation of key fission/fusion genes (e.g., *FIS1*, *DRP1*, *MFN1/2*, *OPA1*), and provided transmission electron microscopy evidence of swollen mitochondria and lipid droplets in salivary epithelium. These findings point to a pervasive mitochondrial defect in SjD. Our present work seeks to advance this understanding by asking a more specific question: is the failure to properly clear these damaged mitochondria—a process known as mitophagy—a key mechanism underlying the progression from mitochondrial stress to overt autoimmune pathology? To address this, we employed a bioinformatic approach to analyze integrated gene expression datasets. Our analysis identified 942 differentially expressed genes (DEGs) in SjD salivary glands. By focusing on mitophagy-related genes within this dataset, we found that five out of 104 genes associated with mitophagy were consistently dysregulated. Although this limited number might suggest that broad activation of the global mitophagy pathway is not a prominent feature in SjD pathogenesis, we propose a more refined explanatory model: the specific dysregulation of a few key regulatory genes may exert a more decisive impact than a wholesale alteration of the pathway. This “critical node” hypothesis is well-supported in systems biology, where the dysfunction of specific bottlenecks or master regulators within complex biological networks can disproportionately disrupt physiological processes [16]. Large-scale functional genomics studies further reinforce this concept, demonstrating that a limited set of key regulators can govern extensive network functions in complex biological systems [17,18]. Our subsequent machine learning analysis—utilizing LASSO regression and random forest algorithms—substantially strengthened this perspective by identifying three central hub genes (*PINK1*, *SQSTM1*, and *GABARAPL1*) from the initial five DEGs. These hub genes demonstrated exceptional diagnostic specificity for SjD, with area under the curve (AUC) values exceeding 0.84 in an independent validation cohort. This finding suggests that the observed transcriptional changes are unlikely to result from random variation but rather reflect a targeted impairment of the core executive machinery of mitophagy. Despite the promising diagnostic performance indicated by ROC analysis (AUCs > 0.84), this finding warrants cautious interpretation due to the relatively limited sample size of the independent validation cohort (GSE173808). While the high AUC values suggest strong discriminatory power, the generalizability and robustness of these biomarkers need to be further confirmed in larger, multi-center, and prospective clinical cohorts. The roles of these genes are well-defined in the canonical mitophagy pathway. *PINK1* (PTEN-induced kinase 1) acts as the primary sensor of mitochondrial health. Upon mitochondrial membrane depolarization, *PINK1* stabilizes on the outer membrane and phosphorylates ubiquitin and the E3 ubiquitin ligase Parkin, marking the mitochondrion for destruction [19,20]. *SQSTM1* (sequestosome 1, best known as p62) then acts as a critical autophagy receptor, binding to ubiquitinated proteins on the damaged organelle and linking it to the LC3/GABARAP family proteins on the forming autophagosome [21,22,23]. *GABARAPL1* itself, a member of this ATG8 family, is essential for the maturation and fusion of autophagosomes with lysosomes [22,24]. The significant dysregulation of this core machinery in SjD provides strong transcriptional evidence for a breakdown in the vital process of mitochondrial quality control. It suggests that the swollen, dysfunctional mitochondria observed by Li et al. may persist, in part, due to a failure in their targeted removal.

The next critical issue pertains to the immunological consequences of mitophagic defects. Our immune cell infiltration analysis yielded results that aligned with and extended the findings of Li et al. [5] who confirmed a severely disrupted immune landscape in SjD salivary glands, characterized by an expansion of effector populations, such as activated CD4+ T cells, CD8+ T cells, follicular helper T cells, plasma cells, and pro-inflammatory M1 macrophages, concomitant with a reduction in regulatory subsets, such as Tregs and anti-inflammatory M2 macrophages. The novel insight from our study comes from correlation analysis, which directly linked the expression of mitophagy hub genes to these specific immune populations. The significant positive correlations with plasma cells and Tregs and the negative correlations with activated CD4+ T cells, NK cells, and M1 macrophages suggest that functional mitophagy is necessary to maintain immune equilibrium. Efficient mitophagy ensures a healthy mitochondrial network that is capable of supporting this metabolic demand. Conversely, mitophagy failure leads to the build-up of damaged ROS-producing mitochondria, creating a hostile metabolic environment that impairs Treg function and promotes the differentiation and activation of inflammatory effector cells, which are more glycolytic. This may explain the negative correlation with hyperactive immune cells. Therefore, defective mitophagy may disrupt the delicate balance between tolerance and inflammation by altering the metabolic fitness of key immune cell subtypes. A study by Kurien et al. [25] not only reported mitochondrial dysfunction in T cells from SjD patients but also identified a distinct “mitophagic transcriptional signature,” reinforcing that impaired mitophagy is a relevant pathophysiological feature in SjD. Notably, the correlation observed in their study between T cell metabolic deficits and patient fatigue further underscores the clinical relevance of this mechanism. not only reported mitochondrial dysfunction in T cells from SjD patients, but also identified a distinct “mitophagic transcriptional signature,” reinforcing that impaired mitophagy is a relevant pathophysiological feature in SjD. Notably, the correlation observed in their study between T cell metabolic deficits and patient fatigue further highlights the clinical relevance of this mechanism.

This proposed mechanism is supported by research on other autoimmune conditions, particularly systemic lupus erythematosus (SLE), in which the role of mitophagy has been more extensively characterized. Mitochondrial dysfunction is a hallmark of SLE in T cells and is characterized by enlarged (megalo) mitochondria and depleted ATP pools. A key driver of this pathology is suppressed mitophagy, often linked to the overexpression of RAB4A, which promotes the degradation of the fission protein DRP1, thereby preventing the isolation of damaged mitochondrial components for removal [26,27]. The resulting accumulation of mitochondrial ROS (mtROS) exacerbates this problem by upregulating RAB4A, creating a vicious cycle of mitochondrial failure that promotes autoimmunity. Similarly, in SLE neutrophils, defective mitophagy leads to the retention and extrusion of oxidized mitochondrial DNA (mtDNA) [28]. Oxidized mtDNA acts as a potent damage-associated molecular pattern (DAMP), triggering the cGAS-STING pathway and fueling the production of type I interferons and autoantibodies, a pathway that is strongly implicated in SjD pathogenesis. The conservation of this mechanism across diseases highlights the fundamental principle that the failure to clear damaged mitochondria generates immunogenic debris that can initiate and perpetuate systemic autoimmunity.

Genetic models provide direct evidence for strengthening causal links. Autophagy-related genes, such as ATG5, ATG7, and IRGM, are associated with autoimmune susceptibility [29]. Most compellingly, naïve Irgm1-/-mice develop a phenotype strikingly reminiscent of SjD, exhibiting lymphocytic infiltration of the salivary and lacrimal glands along with elevated autoantibodies [30]. The deficiency in IRGM1, a regulator of autophagy, leads to type I interferonopathy driven by the release of mtDNA from damaged mitochondria into the cytoplasm, where it activates the cGAS-STING pathway [31,32,33]. Moreover, recent research has established that lactate accumulation, a key metabolic alteration in SjD, initiates this cascade by damaging mitochondria and promoting mtDNA release, which in turn activates cGAS-STING signaling [34].

In addition to its role in preventing DAMP release, mitophagy is integral to the normal development and function of immune cells. The transition of thymocytes to naïve T cells and the maturation of memory NK and CD8+ T cells require a reduction in mitochondrial mass via mitophagy [35,36], which is essential for the survival and function of long-lived, quiescent memory populations [37]. The metabolic shift underlying immune cell polarization, with Tregs and M2 macrophages relying on OXPHOS, and Th1 cells and M1 macrophages relying on glycolysis, is heavily influenced by mitochondrial health, which helps maintain mitophagy [38]. Studies have shown that inhibiting mitophagy promotes a pro-inflammatory M1 macrophage phenotype, whereas inducing it favors an anti-inflammatory M2 state [39]. Therefore, the mitophagic deficiency suggested by our data could fundamentally disrupt the metabolic reprogramming necessary for the proper differentiation and function of critical regulatory immune subsets in SjD. Integrating the broader mitochondrial dysfunction identified by Li et al. [5] with our specific mitophagy signature could provide a powerful new framework for patient stratification and potentially predict metabolic status and guide targeted therapies.

Our study has some limitations. A primary methodological limitation is the computational nature of immune infiltration analysis. Our estimates of immune cell abundance were derived from bulk transcriptomic data using single-sample gene set enrichment analysis (ssGSEA), a widely applied but indirect gene set scoring method. Although such in silico approaches are invaluable for hypothesis generation and for exploring the immune landscape, they are inferential and do not directly measure cell counts. Their accuracy depends on the quality and specificity of the reference gene signatures used and may not fully capture the complexity of cellular states, spatial organization, or the presence of rare cell populations within the tissue microenvironment. Therefore, the correlations observed between hub gene expression and estimated immune cell infiltration should be interpreted as reflecting strong transcriptional associations rather than definitive proof of direct cellular interactions. These computational findings require validation using orthogonal experimental techniques, such as flow cytometry, immunohistochemistry, or single-cell RNA sequencing of matched clinical samples, to confirm the actual composition and functional state of the immune infiltrate. Second, bulk transcriptomic data, although informative, represent a mixture of cell types, making it impossible to definitively attribute the mitophagy signature to specific epithelial or immune cells without single-cell RNA sequencing validation. Future studies should use single-cell technologies to identify the cellular sources of mitophagic dysregulation and validate these findings in larger, well-characterized cohorts.

Despite these limitations, the therapeutic implications of the present study are significant. Recent studies have shown that the pharmacological induction of mitophagy using compounds, such as urolithin A, or agents, such as rapamycin (an mTOR inhibitor), can ameliorate disease pathology in lupus models [26] and influence macrophage polarization [39]. Our identification of specific dysregulated mitophagy hub genes in SjD provides a strong rationale for exploring targeted interventions to restore mitochondrial homeostasis and disrupt the cycle of inflammation. This rationale is strongly supported by recent experimental evidence showing that rapamycin alleviates submandibular gland pathology in an SjD model by enhancing autophagy, which acts as a key brake limiting the activation of the cGAS-STING signaling pathway [40]. Furthermore, pioneering research on natural compounds has identified baicalin and quercetin as effective agents that can reduce mtDNA release and downstream inflammatory responses by specifically modulating the mtDNA-cGAS-STING axis, thereby offering a novel and targeted therapeutic strategy for SjD [41].

In conclusion, our study provides evidence of mitophagy abnormalities in SjD and suggests their potential contribution to disease pathogenesis through their effects on immune cell function and inflammation. The correlation between mitophagy gene expression and specific immune cell populations indicates the potential mechanisms through which mitochondrial quality control may influence the immune dysregulation characteristics of SjD. Although challenges remain in understanding the precise mechanisms and developing targeted therapies, the investigation of mitophagy in SjD opens new avenues for understanding disease pathogenesis and developing novel treatment strategies. Future studies may ultimately lead to improved outcomes in patients with complex autoimmune disorders.

## 4. Materials and Methods

### 4.1. Microarray Data and Mitophagy-Related Gene Dataset

Raw gene expression data for patients with SjD and controls were obtained from the GEO database, specifically from the GSE23117 and GSE40611 datasets. The GSE23117 dataset, which comprises samples from the minor salivary gland profiled using the Affymetrix Human Genome U133 Plus 2.0, included samples from 11 patients with SjD and 4 healthy individuals. The GSE40611 dataset, derived from parotid gland tissue and profiled with the Affymetrix Human Genome U133 Plus 2.0 Array platform, comprised 31 samples from patients with SjD and 18 healthy individuals. Mitophagy-related genes were identified and retrieved from the KEGG pathway database. All genes annotated under the “Mitophagy” pathway (KEGG entry: hsa04137) were included. The gene list was downloaded from the official KEGG website (https://www.kegg.jp/pathway/hsa04137, accessed on 16 November 2024, yielding a total of 104 genes. To maintain objectivity, no additional manual curation (e.g., gene addition or removal) was applied to this list.

### 4.2. Salivary Gland Sample Collection

Salivary gland tissue samples were collected from six patients with SjD and six healthy controls at the First Hospital of Lanzhou University. After the participants provided informed consent, salivary gland samples were obtained. Tissue samples were stored in liquid nitrogen for subsequent experiments.

### 4.3. RNA Extraction and Real-Time Polymerase Chain Reaction

Total RNA was extracted from salivary gland tissue samples using TRIZOL reagent (15596026, Invitrogen, Carlsbad, CA, USA). cDNA was synthesized using the HiScript III RT SuperMix for qPCR kit (+gDNA wiper), according to the manufacturer’s protocol (R323-01, Vazyme, Nanjing, China). A SYBR qPCR kit (Q711; Vazyme, Nanjing, China) was used for PCR. Primer sequences used in this study are listed in Supplementary Table S1. mRNA transcription levels of mitophagy-related hub genes were determined using a Light Cycler 480 instrument (Roche, Basel, Switzerland). The relative transcription levels of mitophagy-related hub genes were analyzed using the 2^−ΔΔCt^ method, compared with β-actin as a reference gene.

### 4.4. Screening of Differentially Expressed Genes in Sjögren’s Disease

The expression profiles of the GSE23117 and GSE40611 datasets were merged into a new microarray dataset. Quality control was performed, including log2 transformation and visualization of data distribution. Batch effects were removed using the ComBat algorithm from the R package “sva.” (version 3.57.0) The effectiveness of batch-effect correction was assessed by comparing the distribution and clustering of the samples before and after processing, as shown in Supplementary Figure S1. Subsequently, DEGs between the salivary gland tissue samples of patients with SjD and the control group were identified using the “limma” package in R (version 3.65.0). Genes with a corrected *p*-value (adj. *p*-value) < 0.05, and |log2(fold change)| > 1.0, were considered as DEGs. Finally, heatmaps, volcano plots, and boxplots were created using the R packages “pheatmap” (version 1.0.13) (or “ComplexHeatmap” (version 2.26.0)) and “ggplot2” (version 4.0.2).

### 4.5. Functional Analysis of Differentially Expressed Genes

GO and KEGG pathway enrichment analyses were performed using the ‘clusterProfiler’ package (v4.1.1) in R. Enrichment significance was assessed using the hypergeometric test with Benjamini–Hochberg false discovery rate (FDR) correction. Terms with an FDR-adjusted *p*-value < 0.05 were considered statistically significant. GeneRatio was calculated as follows: (number of DEGs in a term)/(total number of background genes annotated to that term).

### 4.6. PPI Analysis of Mitophagy-Related Differentially Expressed Genes

The PPI network was constructed using the STRING and GeneMANIA databases to assess interactions among the five differentially expressed mitophagy-related genes.

### 4.7. Identification of Hub Genes and ROC Curve Analysis

Two machine learning algorithms, the LASSO and Random Forest, were used to screen for hub genes. The LASSO algorithm was executed using the “glmnet” package (version 4.1.10), whereas the Random Forest algorithm was implemented with the “random Forest” package (version 4.7.1.2). Genes identified using both methods were intersected and further intersected with previously identified mitophagy-related DEGs to determine hub genes associated with mitophagy. Correlation analysis of the three mitophagy-related hub genes was conducted using the “circlize” package (version 0.4.16). ROC curve analysis of the hub genes was conducted using the “pROC” package in the validation dataset GSE173808, which comprises samples from labial salivary gland tissue profiled with the Illumina HiSeq high-throughput sequencing (RNA-Seq) platform, to evaluate their specificity and diagnostic value for SjD. The AUC value was quantified, with an AUC > 0.6 considered statistically significant.

### 4.8. Immune Infiltration Analysis

Single-sample gene set enrichment analysis (ssGSEA) was used to quantitatively assess the infiltration of immune cells into each salivary gland sample in the microarray dataset. The “ggplot2” package was used to detect and visualize the relationship between hub genes and the abundance of 22 types of immune cells, with a statistical significance level set at *p* < 0.05.

### 4.9. Reactome-Based Gene Set Enrichment Analysis

GSEA was performed to investigate the enriched biological signals associated with the three mitophagy-related hub genes. The normalized enrichment score (NES) was used to evaluate the strength of enrichment, with values > 0 indicating a positive correlation between the gene and pathway.

### 4.10. Prediction of miRNAs and Transcription Factors for Hub Genes

The Reg Network database (https://regnetworkweb.org/, accessed on 25 December 2024) was used to predict the upstream miRNAs and transcription factors for the three mitophagy-related hub genes, and the results were visualized using Cytoscape (version 3.10.3).

### 4.11. Prediction of Target Compounds

Network Analyst is a comprehensive web-based application that facilitates the design of customized networks for pharmacogenomic research. In this study, a Network Analyst was used to establish a protein-compound network.

### 4.12. Statistical Analysis

Statistical analyses were conducted using R software (version 4.1.1) and GraphPad Prism 5. qRT-PCR assays were performed in triplicate or more. Data analysis was performed using GraphPad Prism (version 9.4.1, GraphPad Software, San Diego, CA, USA), and numerical data are presented as bars. To identify differences between groups, Student’s *t*-test was used as the statistical method, with a significance level set at *p* < 0.05.

## 5. Conclusions

We identified three mitophagy-related hub genes and demonstrated their correlation with the immune landscape in SjD. These results implicate mitophagic processes in SjD pathogenesis, which may be associated with immune infiltration. This study provides a new perspective for understanding SjD and a rationale for exploring novel treatment options.

## Data Availability

The datasets (GSE23117, GSE40611 and GSE173808) for this study can be found in the GEO database (GSE23117: https://www.ncbi.nlm.nih.gov/gds/?term=GSE23117, accessed on 30 October 2024; GSE40611: https://www.ncbi.nlm.nih.gov/gds/?term=GSE40611, accessed on 30 October 2024; GSE173808: https://www.ncbi.nlm.nih.gov/gds/?term=GSE173808, accessed on 30 October 2024).

## Supplementary Materials

The following supporting information can be downloaded at https://www.mdpi.com/article/10.3390/ijms27083365/s1.
