# Supplementary material for: Mitophagy and Immune Infiltration in Primary Sjögren’s Disease: Insights from Bioinformatics Analysis
Source: Int J Mol Sci. 2026 Apr 9;27(8):3365. doi: 10.3390/ijms27083365 (PMC13116969; doi:10.3390/ijms27083365)
Supplement: Supplementary file 1 [file ijms-27-03365-s001.zip › Figure legends.pdf]

Figure legends

Figure S1 (A) PCA plots showing the expression data of GSE23117 and GSE40611. (B) PCA plots showing the combined microarray set of GSE23117 and GSE40611 with the removal of batch effects. (C) box line blots before homogenization. (D)box line blots after homogenization. (E) volcano map of DEGs. (F) heatmap of the top 20 significantly upregulated or downregulated DEGs.

Figure S2 (A) Identification of DEGs between salivary gland and controls in the combined microarray set of GSE23117 and GSE40611. (B)Venn diagram displayed the overlap of genes screened by two machine learning algorithms

Figure S3 (A) GO analysis of 5 mitophagy-related DEGs. (B) KEGG analysis of 5 mitophagy-related DEGs. (C) correlation between 5 mitophagy-related DEGs and the top five pathways of KEGG.

Figure S4 PPI network of 5 mitophagy-related DEGs constructed by STRING (A) and Genemania (B).

Figure S5 Upstream regulatory miRNAs and transcription factors

Figure S6 Compounds associated with the hub genes to identify those with potential therapeutic effects for Sjögren's disease

Table legends

Supplementary Table S1 The primer sequences used in this study

Supplementary Table S2 Compounds along with their corresponding target genes
