# Supplementary material for: Mitophagy and Immune Infiltration in Primary Sjögren’s Disease: Insights from Bioinformatics Analysis
Source: Int J Mol Sci. 2026 Apr 9;27(8):3365. doi: 10.3390/ijms27083365 (PMC13116969; doi:10.3390/ijms27083365)
Supplement: Supplementary file 1 [file ijms-27-03365-s001.zip › Supplementary Table S2.pdf]

Supplementary Table S2. Primes sequences for qRT-PCR

	Sequence (5'- 3')	*Sequence (5'- 3')*
Primer Set	*Forward*	*Reverse*
PINK1	CCATCGGGATCTCAAGTCCG	GATCACTAGCCAGGGACAGC
SQSTM1	TGT GTA GCG TCT GCG AGG GAA A	AGT GTC CGT GGT TCA CCT TCC G
GABARAPL1	ATCCCTCCCACCAGTGCTAC	ATAACACCTTCTGCCCCACTTCTC
