# Supplementary figures and images for: Mitophagy and Immune Infiltration in Primary Sjögren’s Disease: Insights from Bioinformatics Analysis
Source: Int J Mol Sci. 2026 Apr 9;27(8):3365. doi: 10.3390/ijms27083365 (PMC13116969; doi:10.3390/ijms27083365)

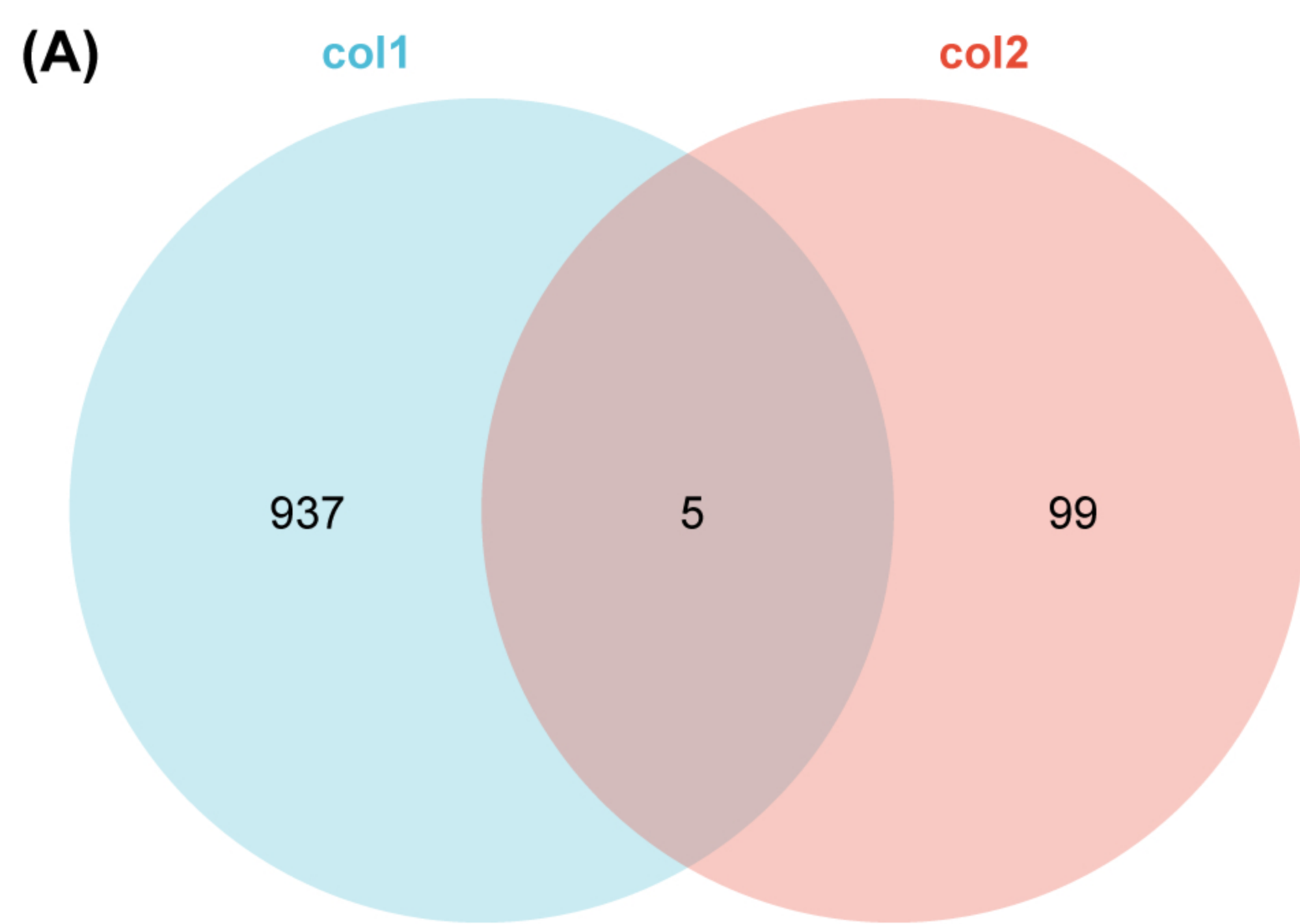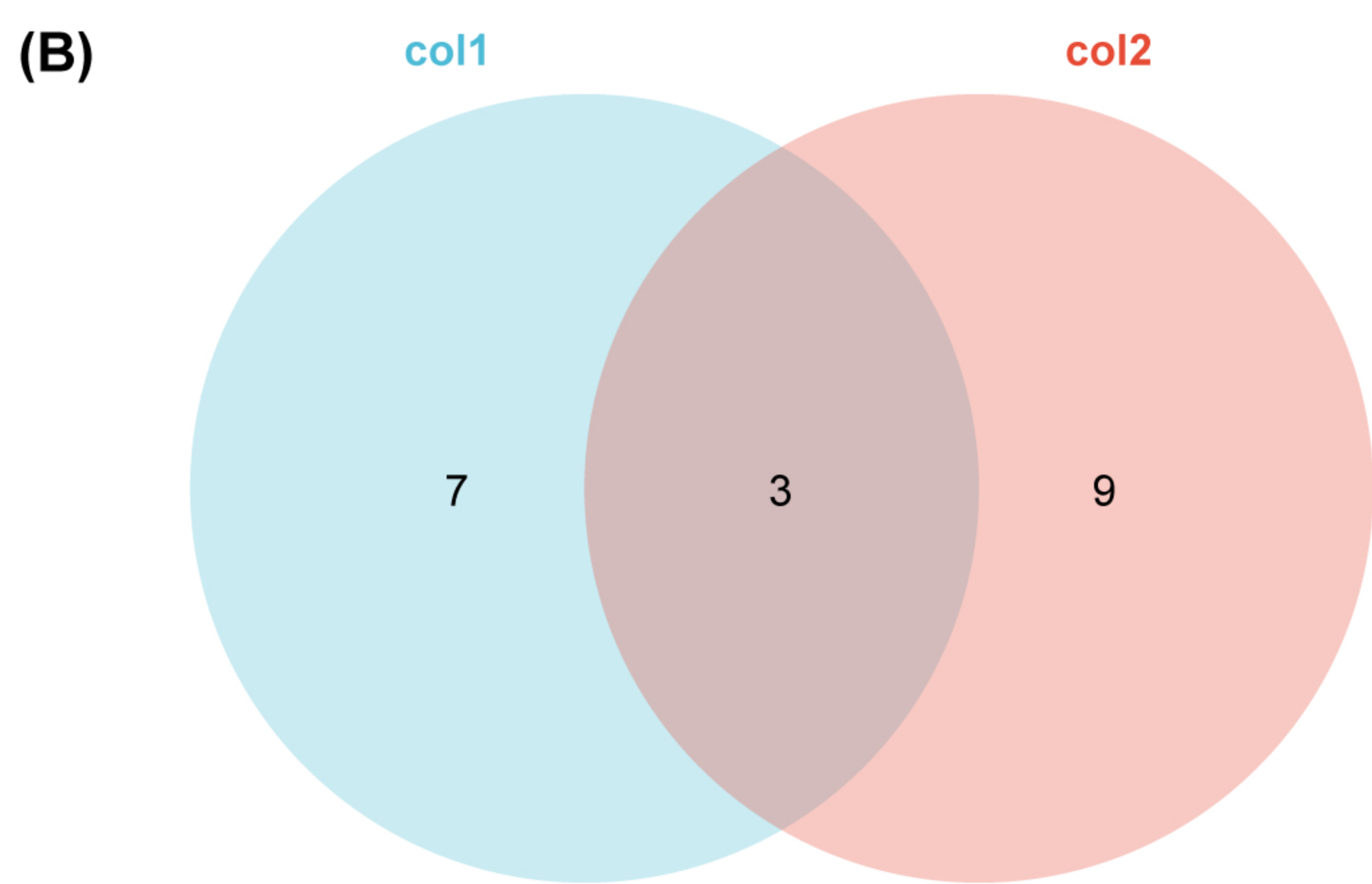

Supplement: Supplementary file 1 [file ijms-27-03365-s001.zip › Supplementary Figures/Figure S2.pdf]

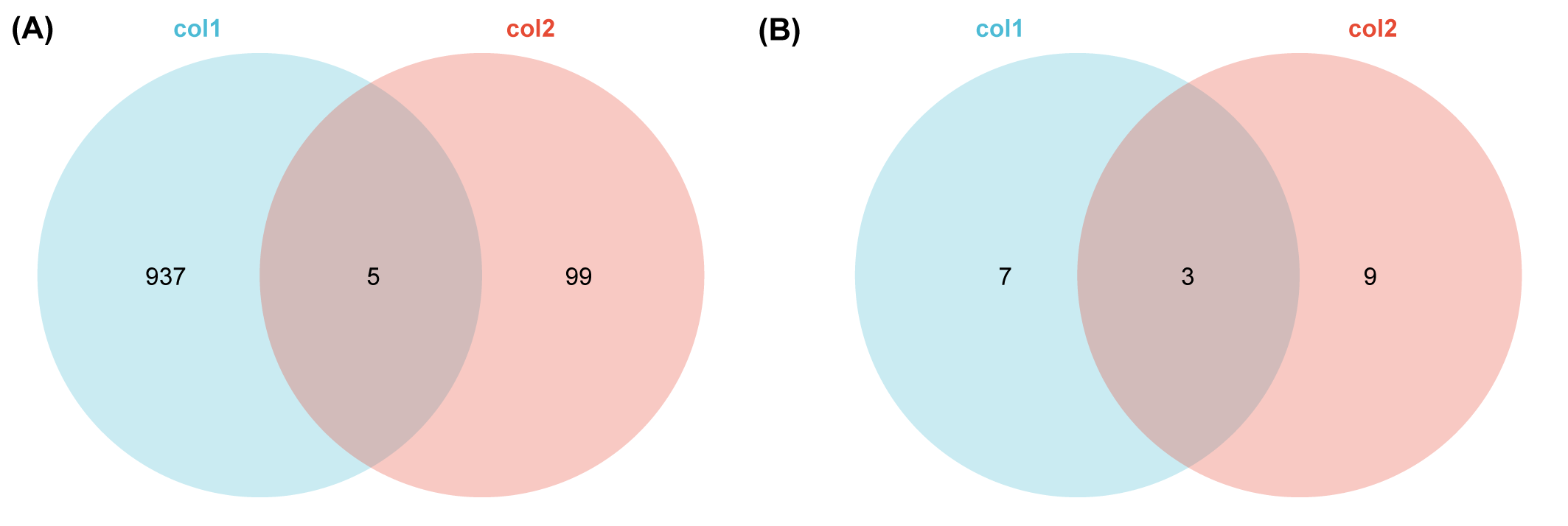

Supplement: Supplementary file 1 [file ijms-27-03365-s001.zip › Supplementary Figures/Figure S2.tif]

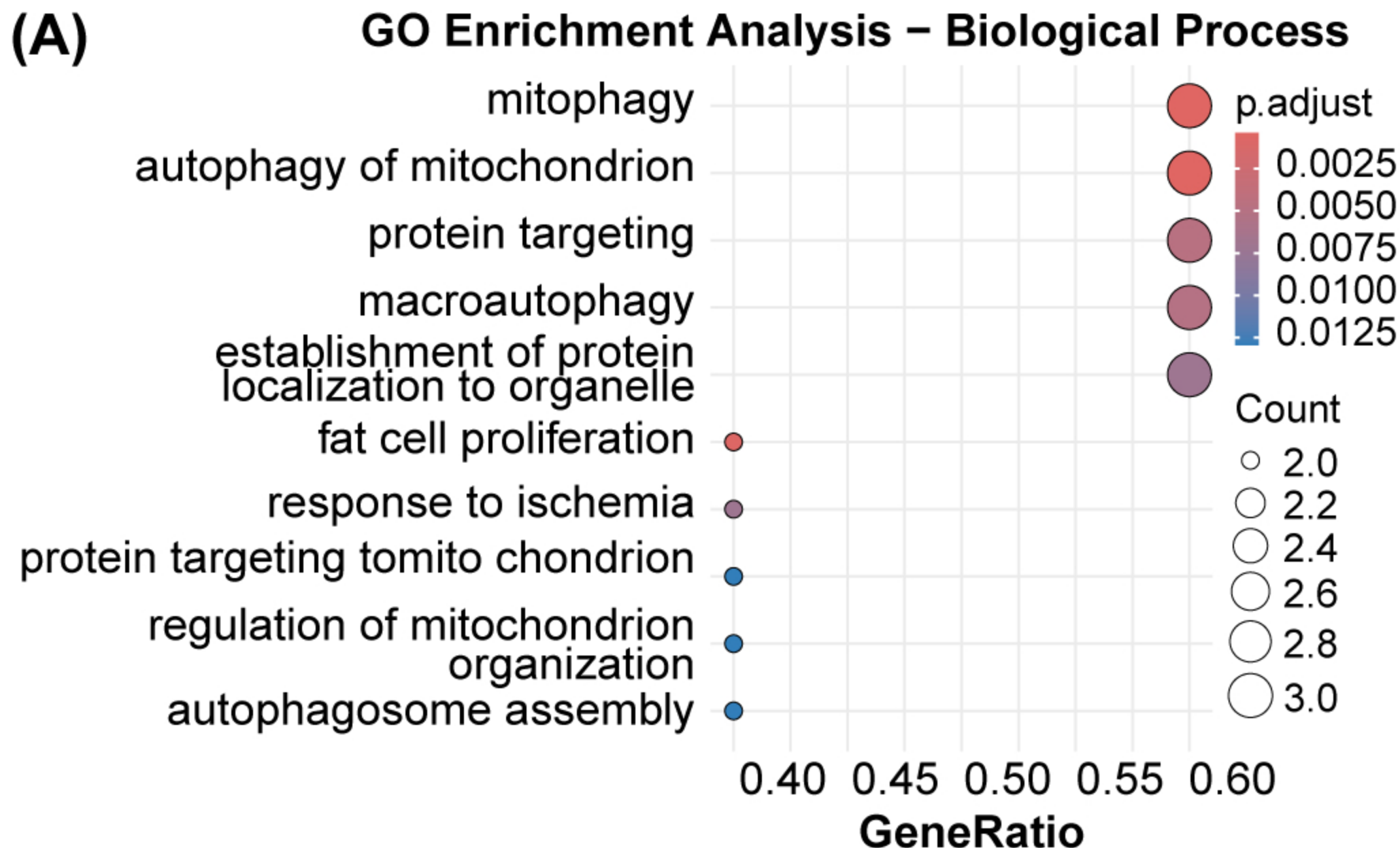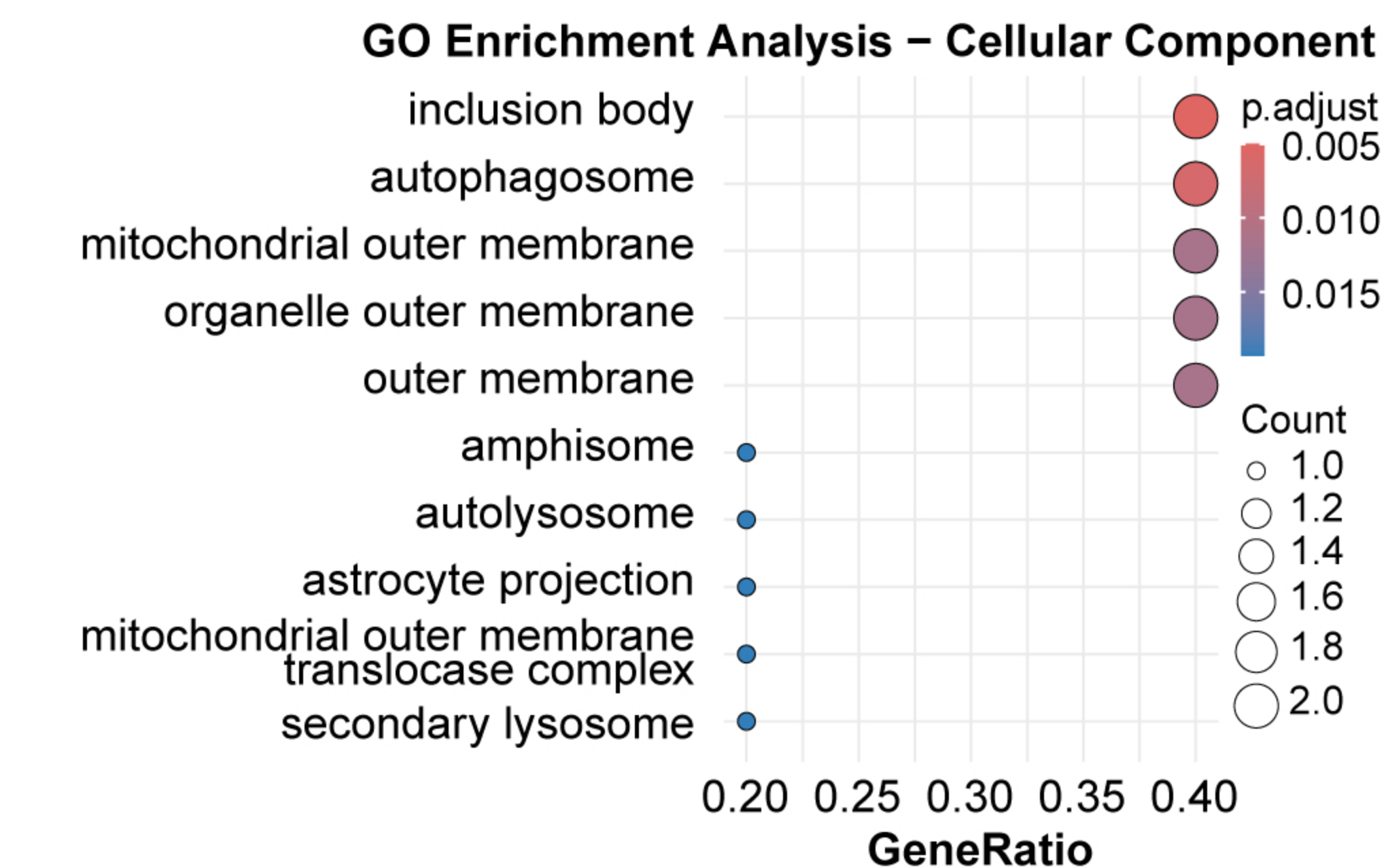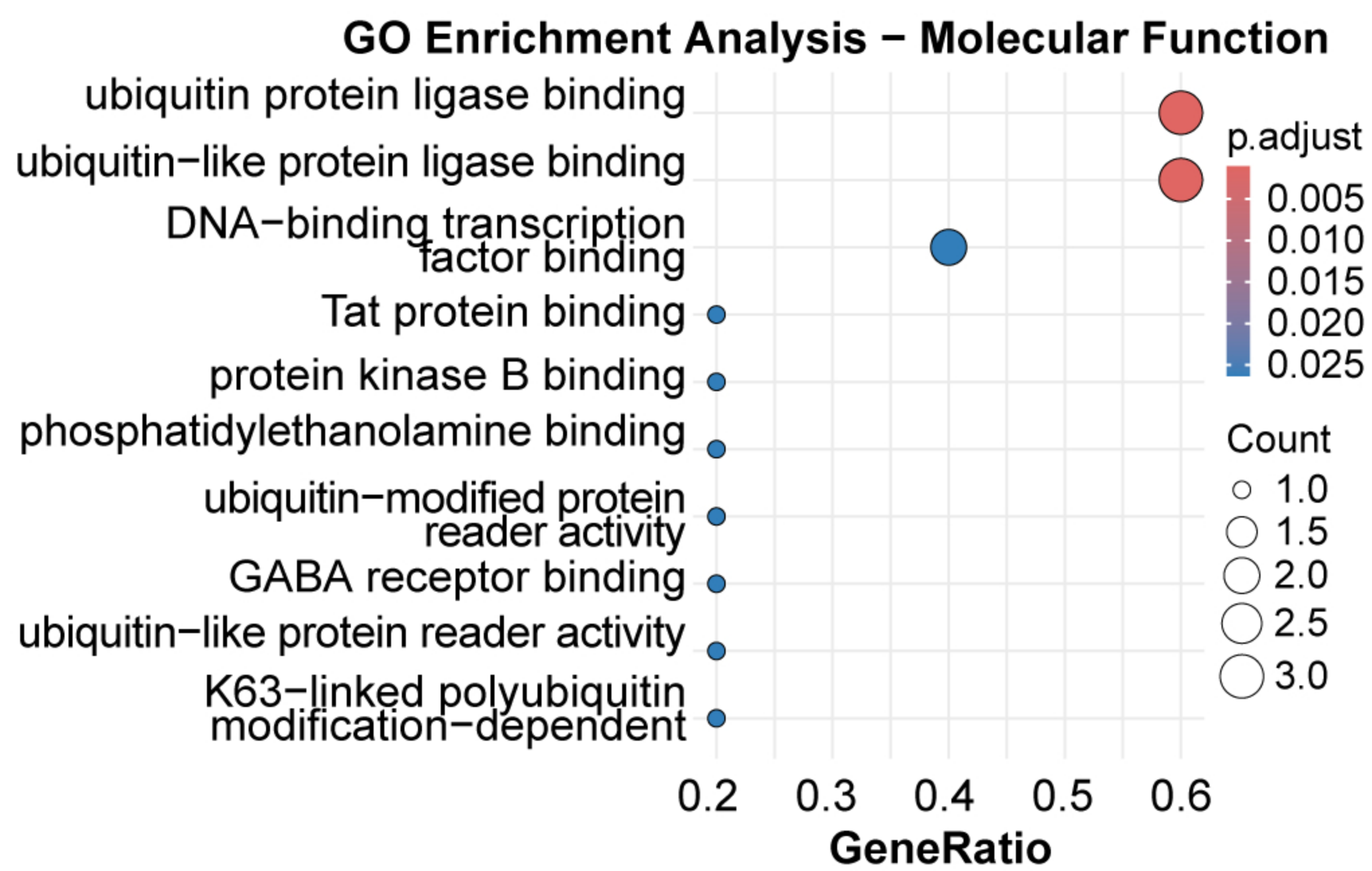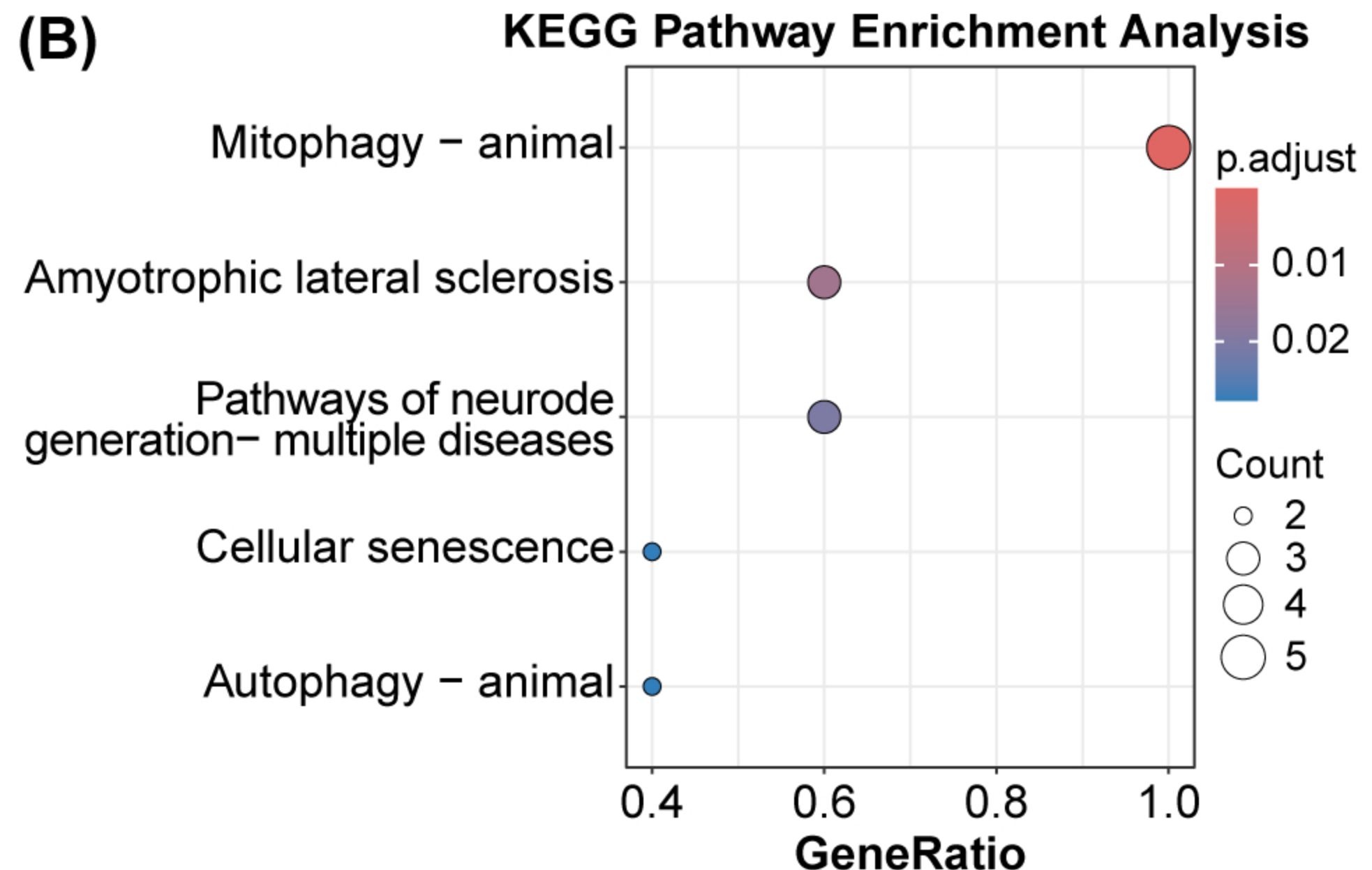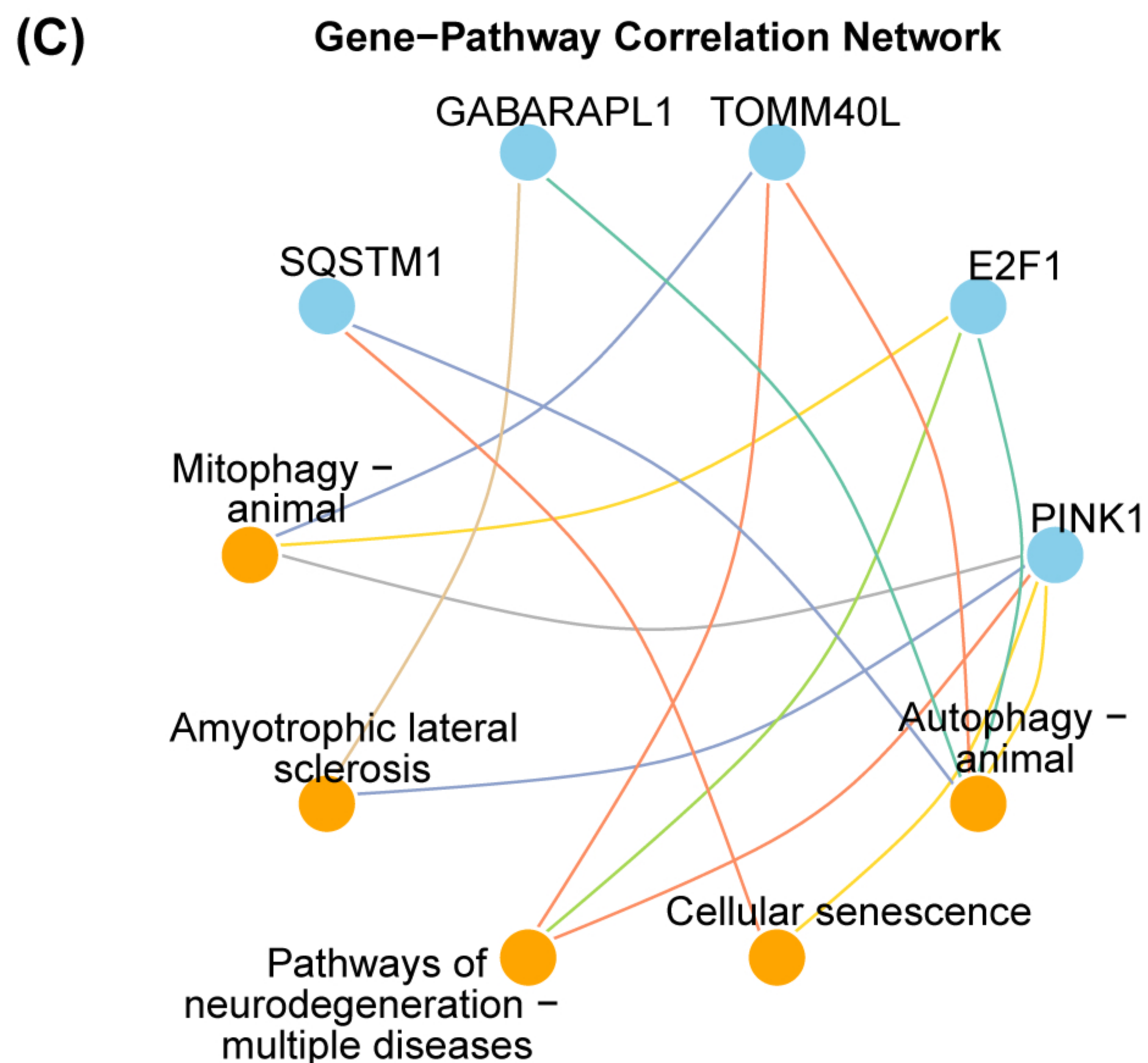

Supplement: Supplementary file 1 [file ijms-27-03365-s001.zip › Supplementary Figures/Figure S3.pdf]

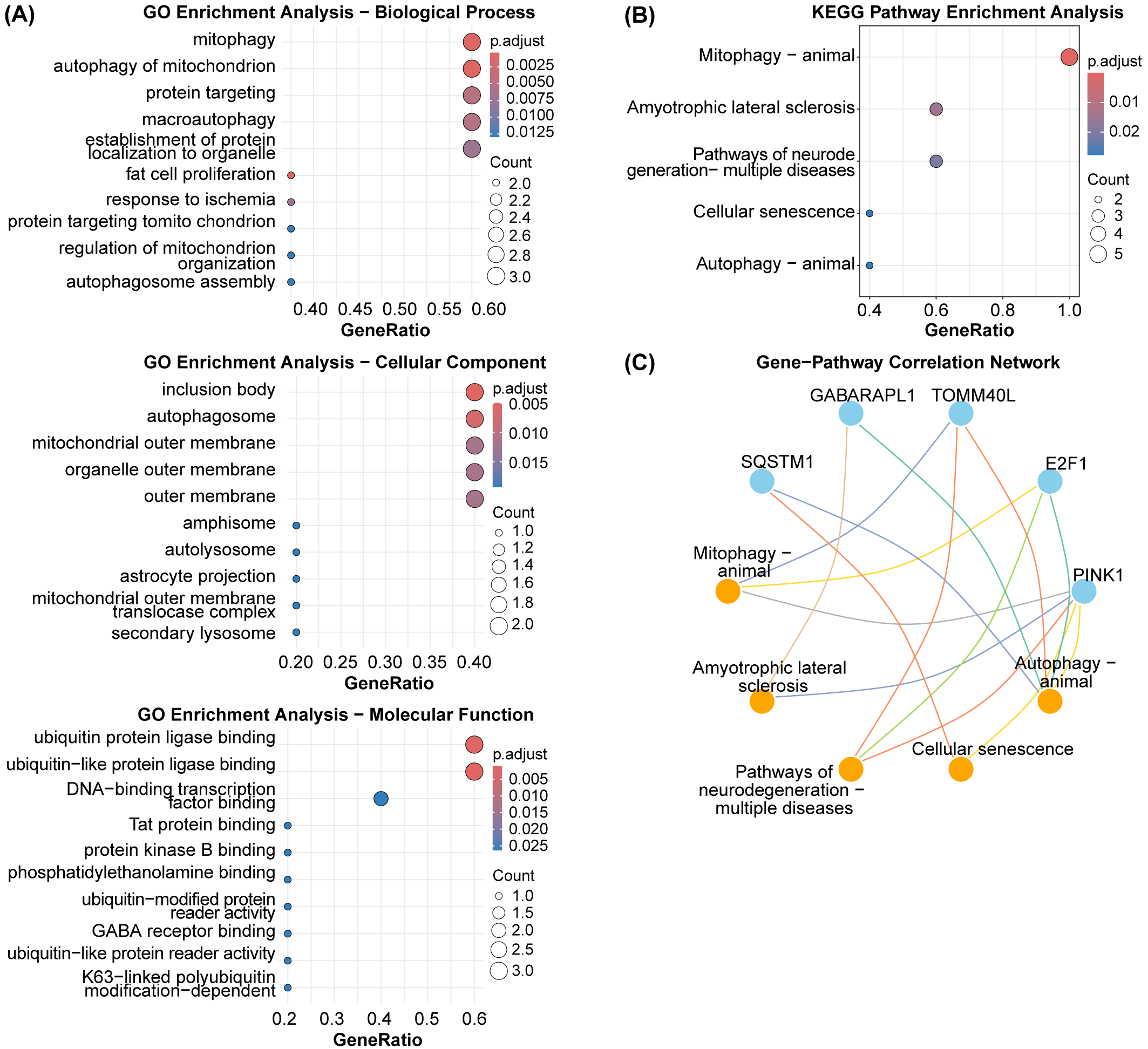

Supplement: Supplementary file 1 [file ijms-27-03365-s001.zip › Supplementary Figures/Figure S3.tif]

**(A)**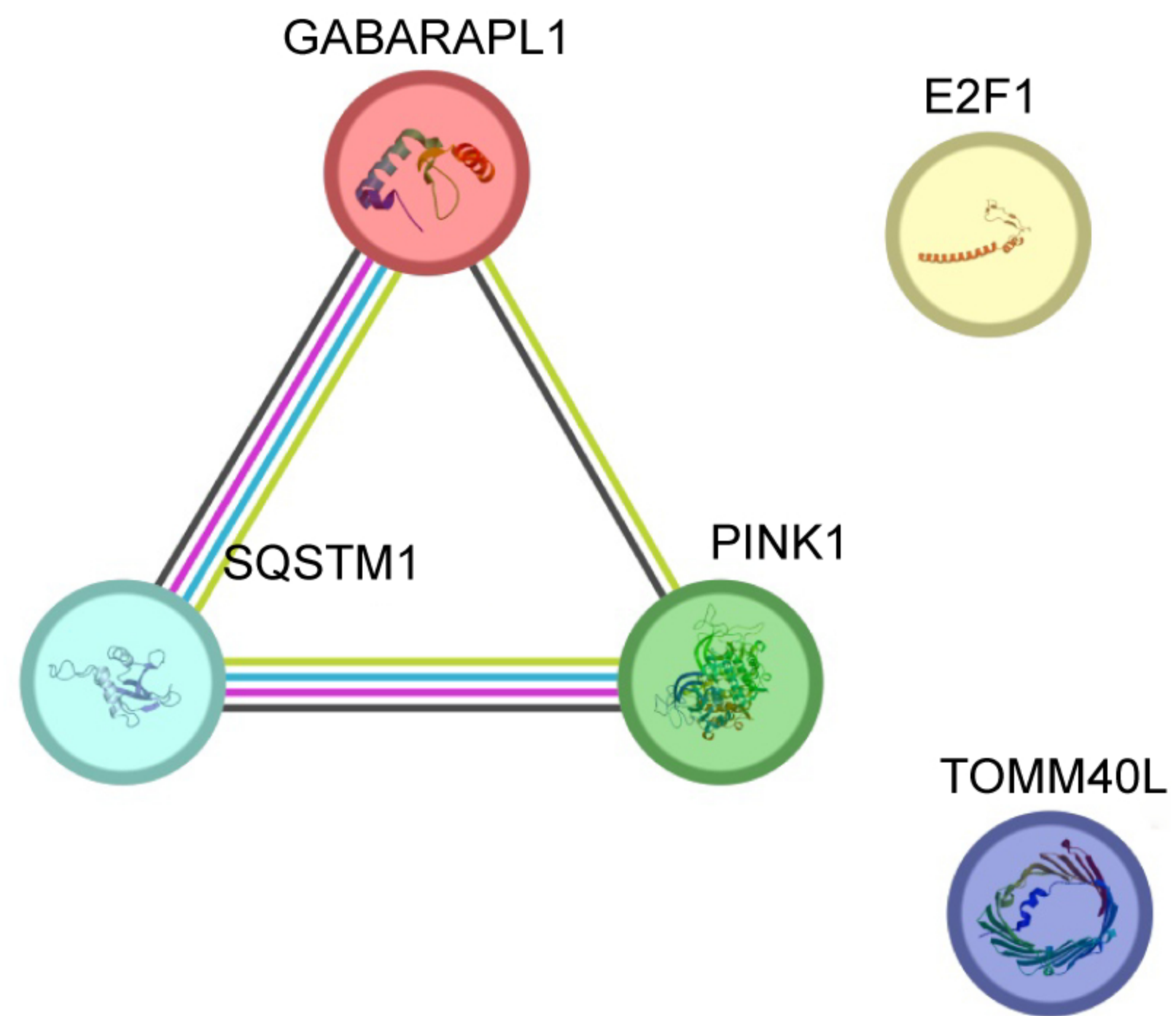**(B)**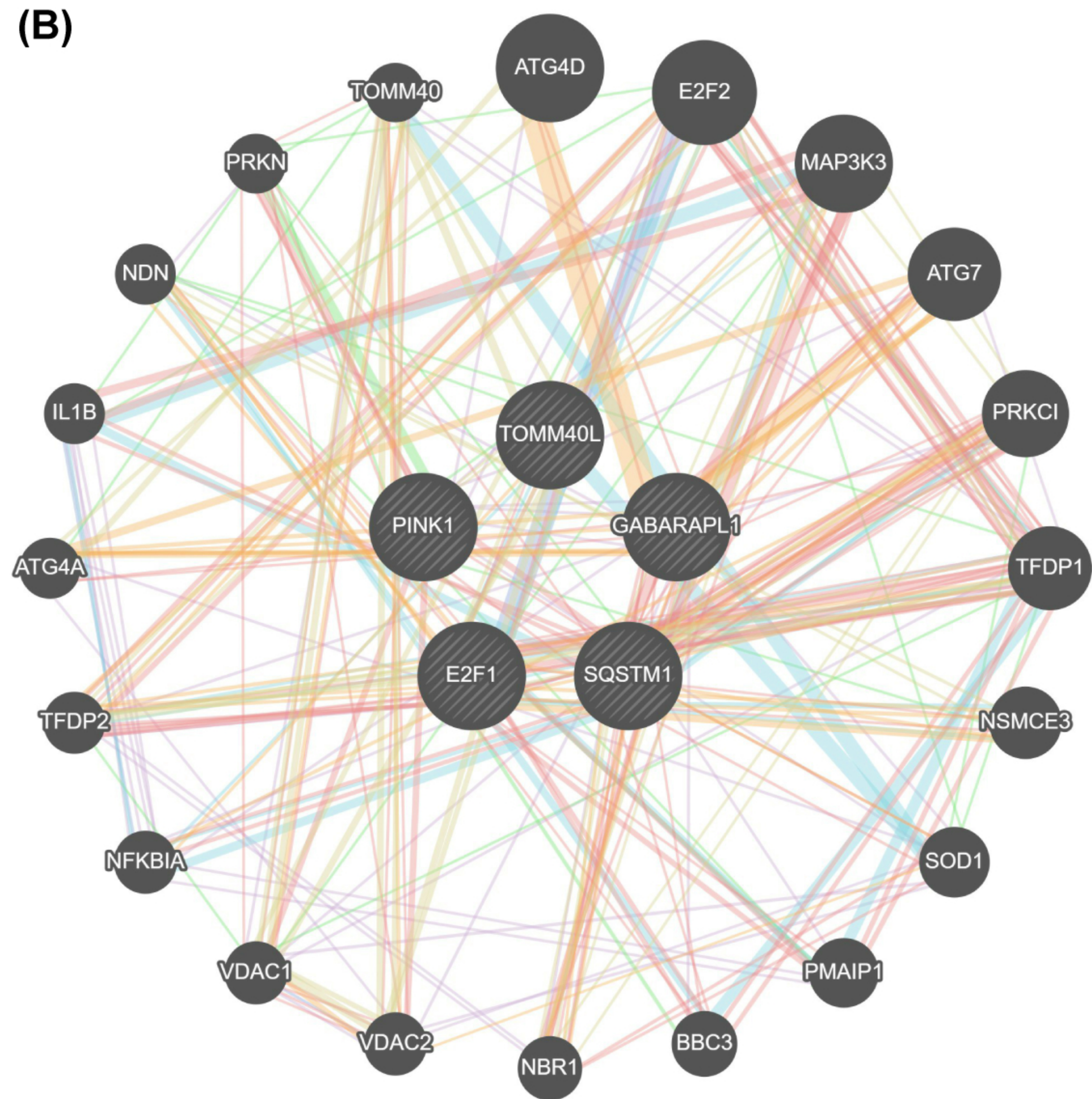

Supplement: Supplementary file 1 [file ijms-27-03365-s001.zip › Supplementary Figures/Figure S4.pdf]

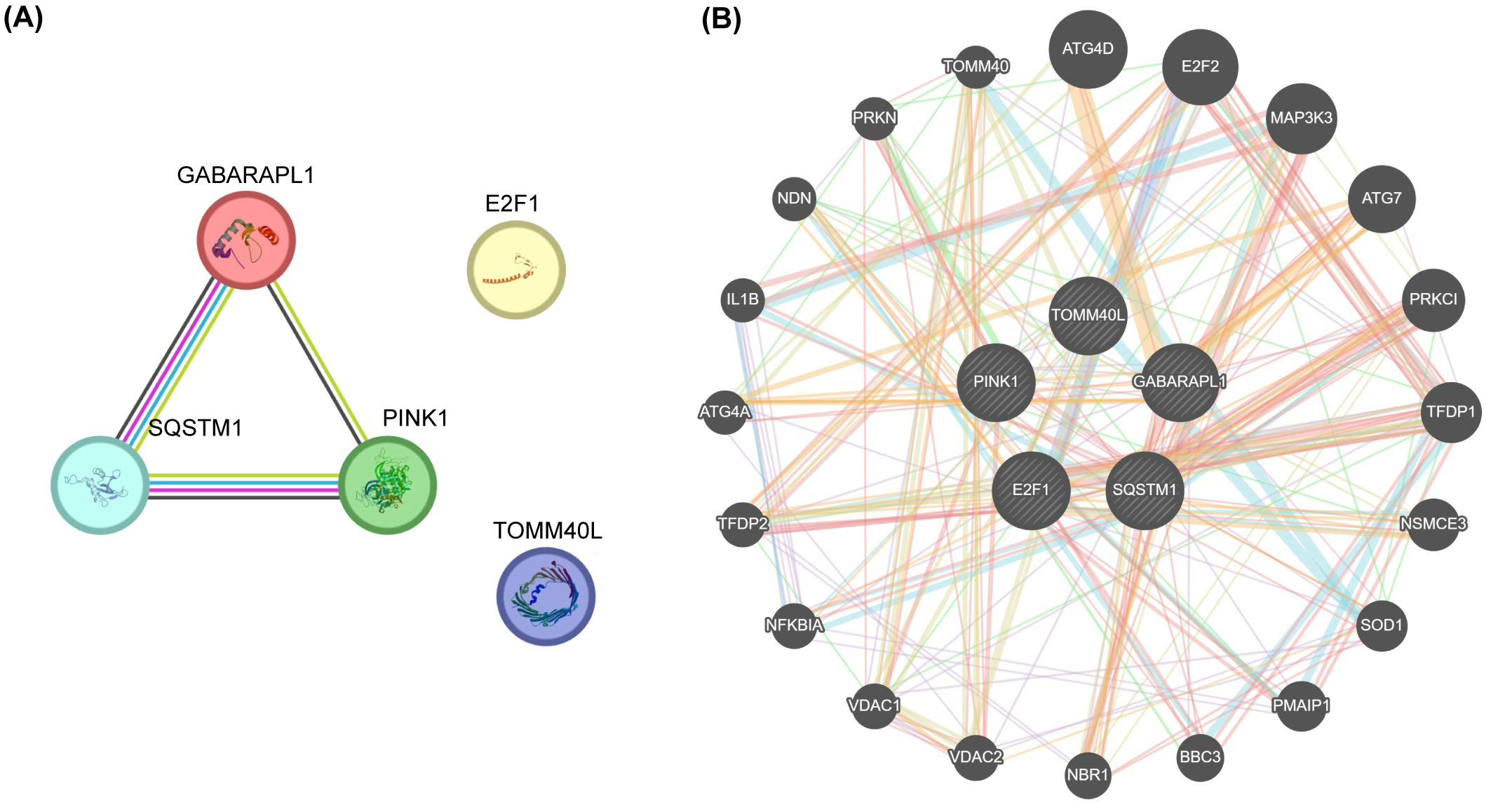

Supplement: Supplementary file 1 [file ijms-27-03365-s001.zip › Supplementary Figures/Figure S4.tif]

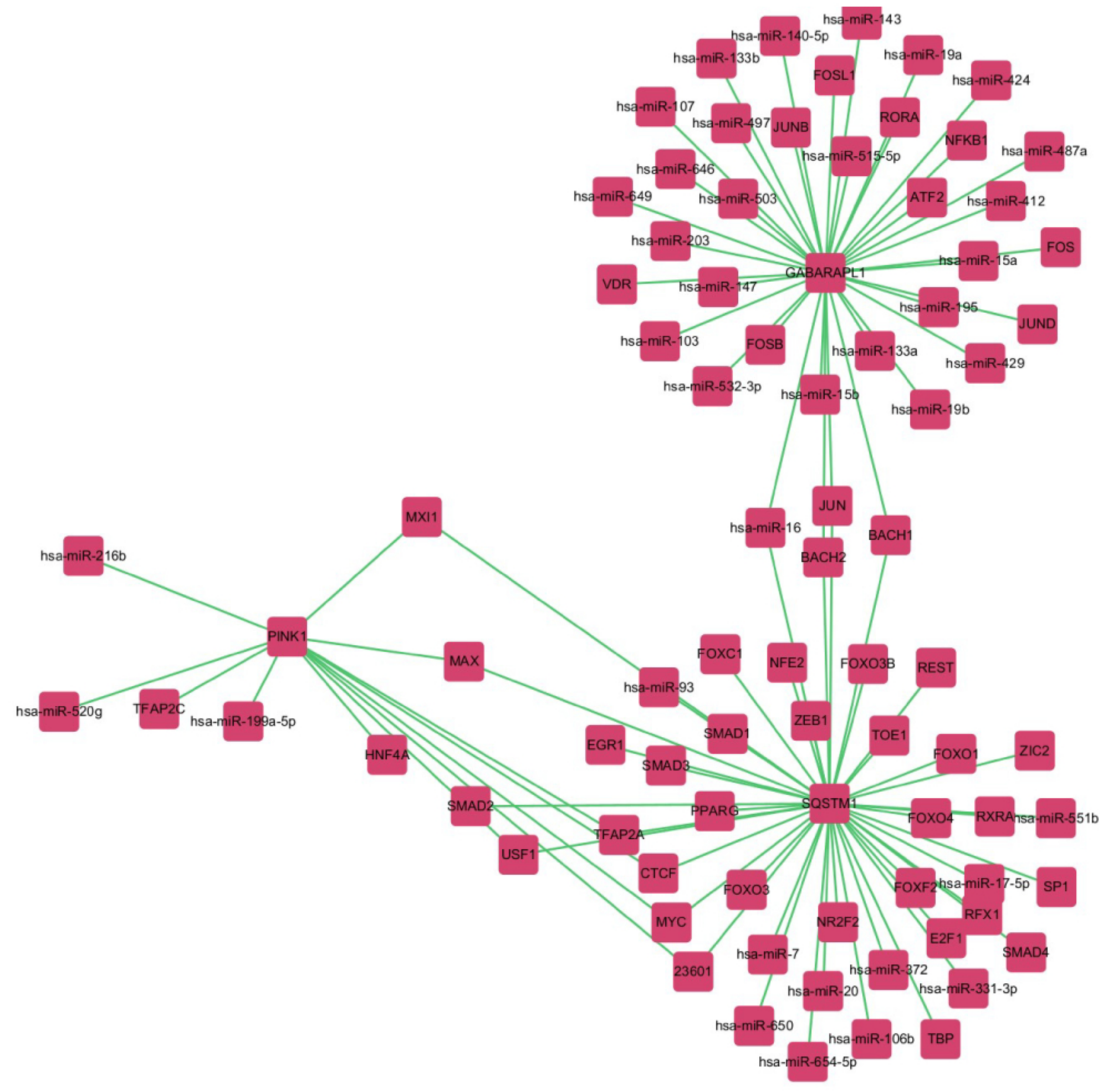

Supplement: Supplementary file 1 [file ijms-27-03365-s001.zip › Supplementary Figures/Figure S5.pdf]

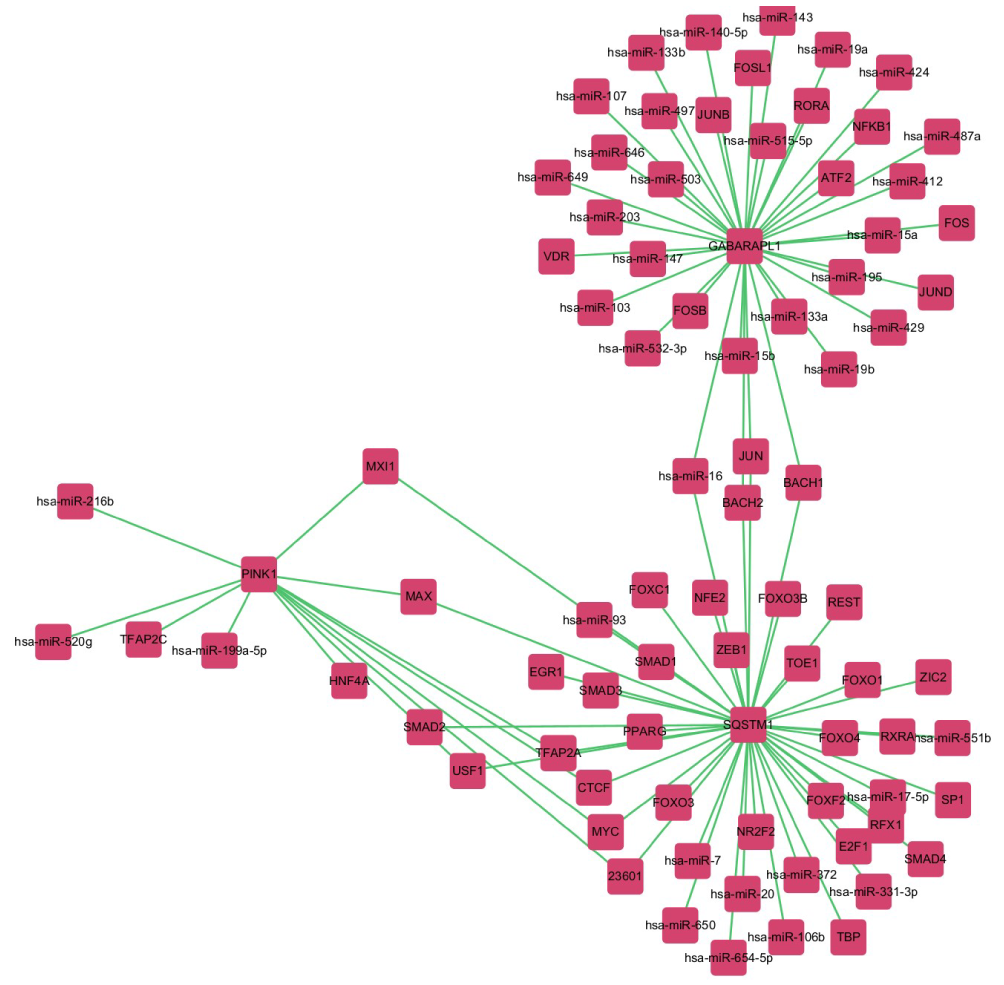

Supplement: Supplementary file 1 [file ijms-27-03365-s001.zip › Supplementary Figures/Figure S5.tif]

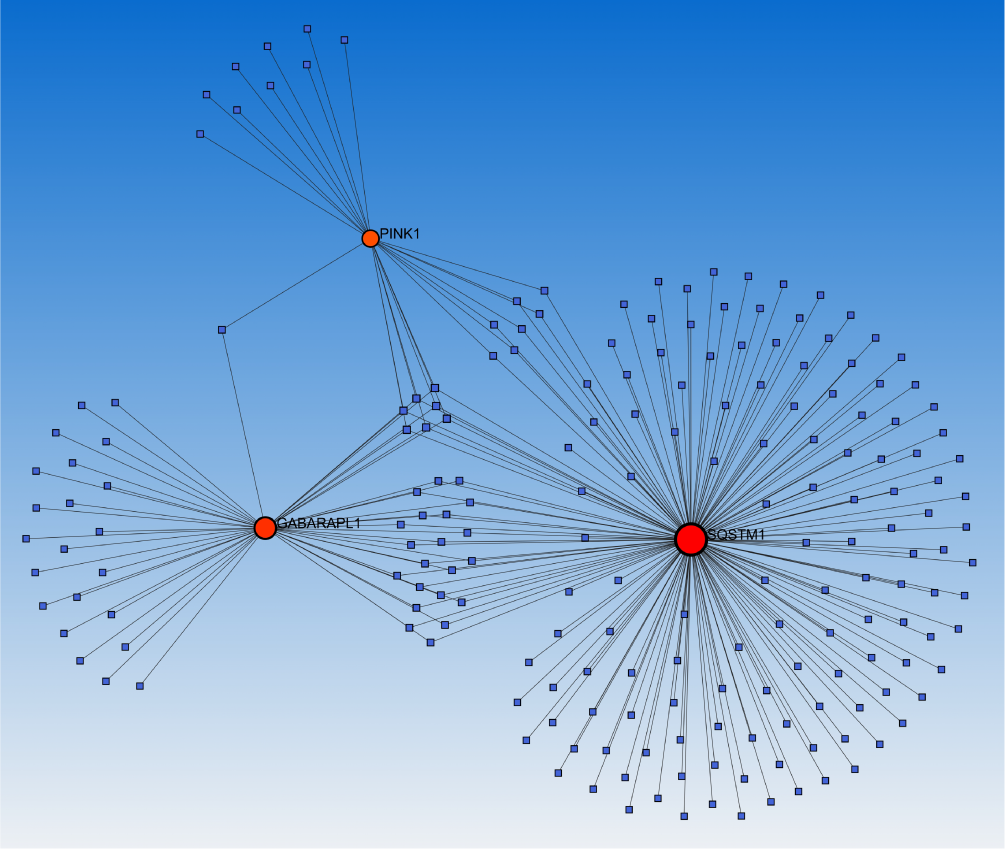

Supplement: Supplementary file 1 [file ijms-27-03365-s001.zip › Supplementary Figures/Figure S6.tif]

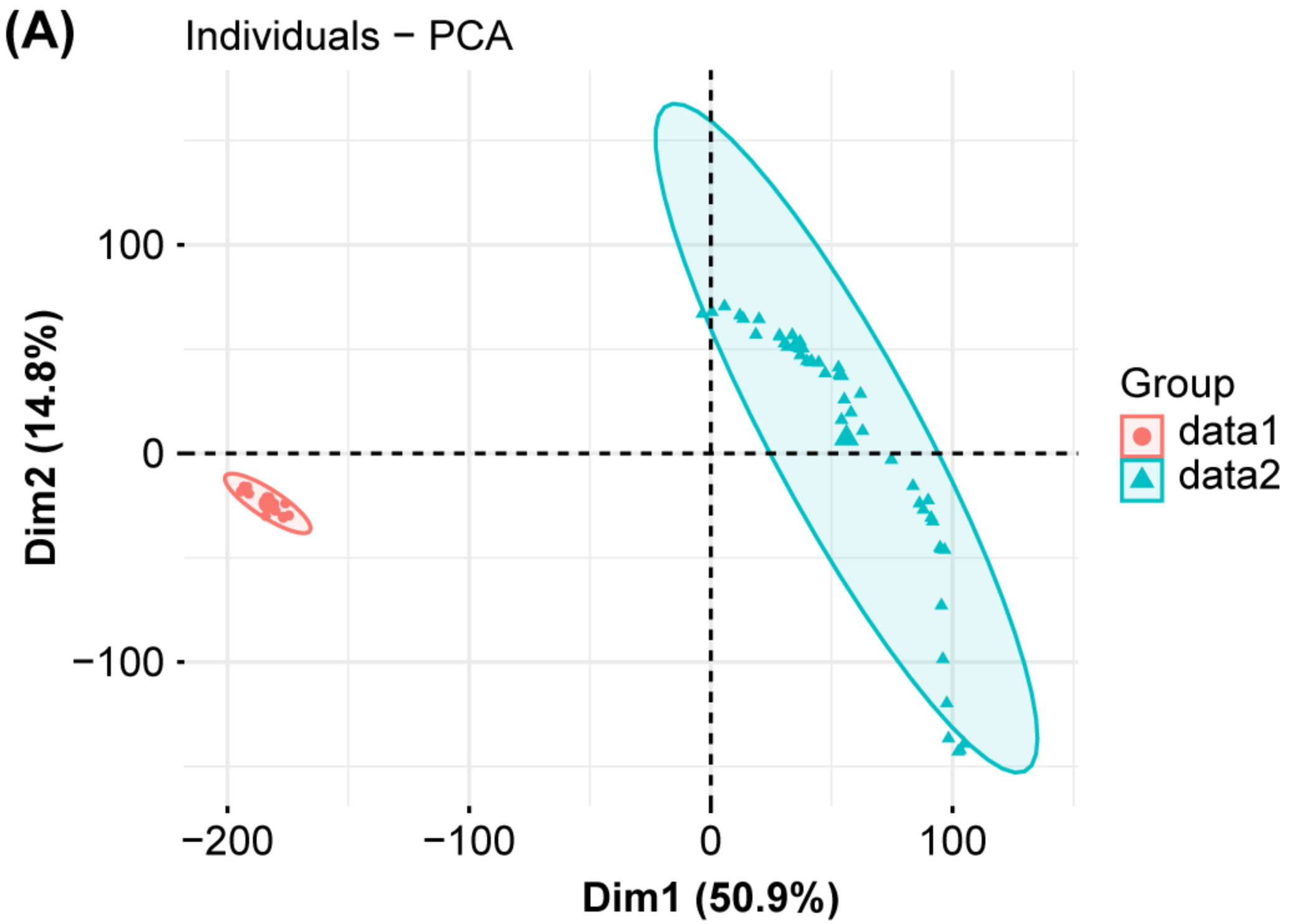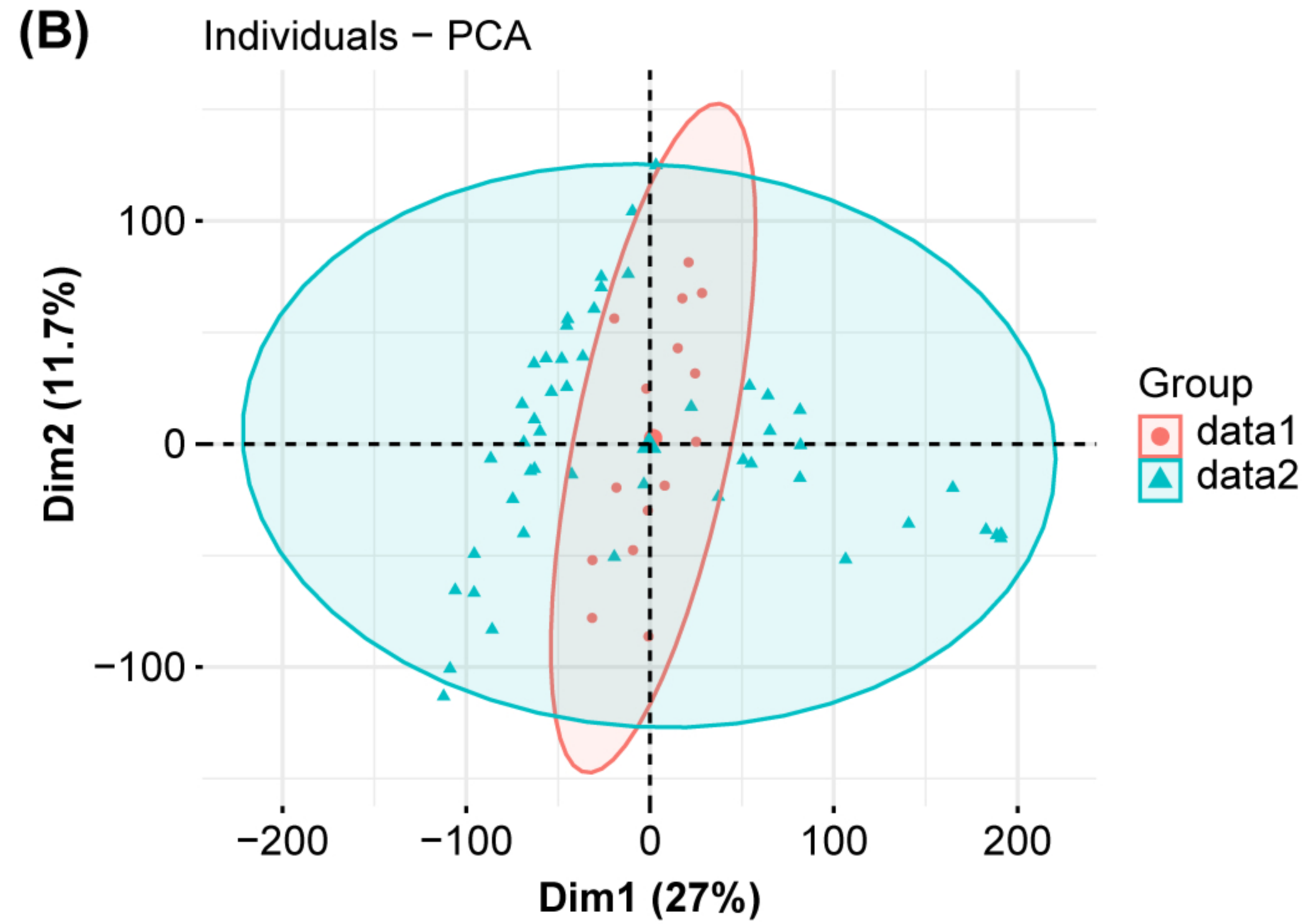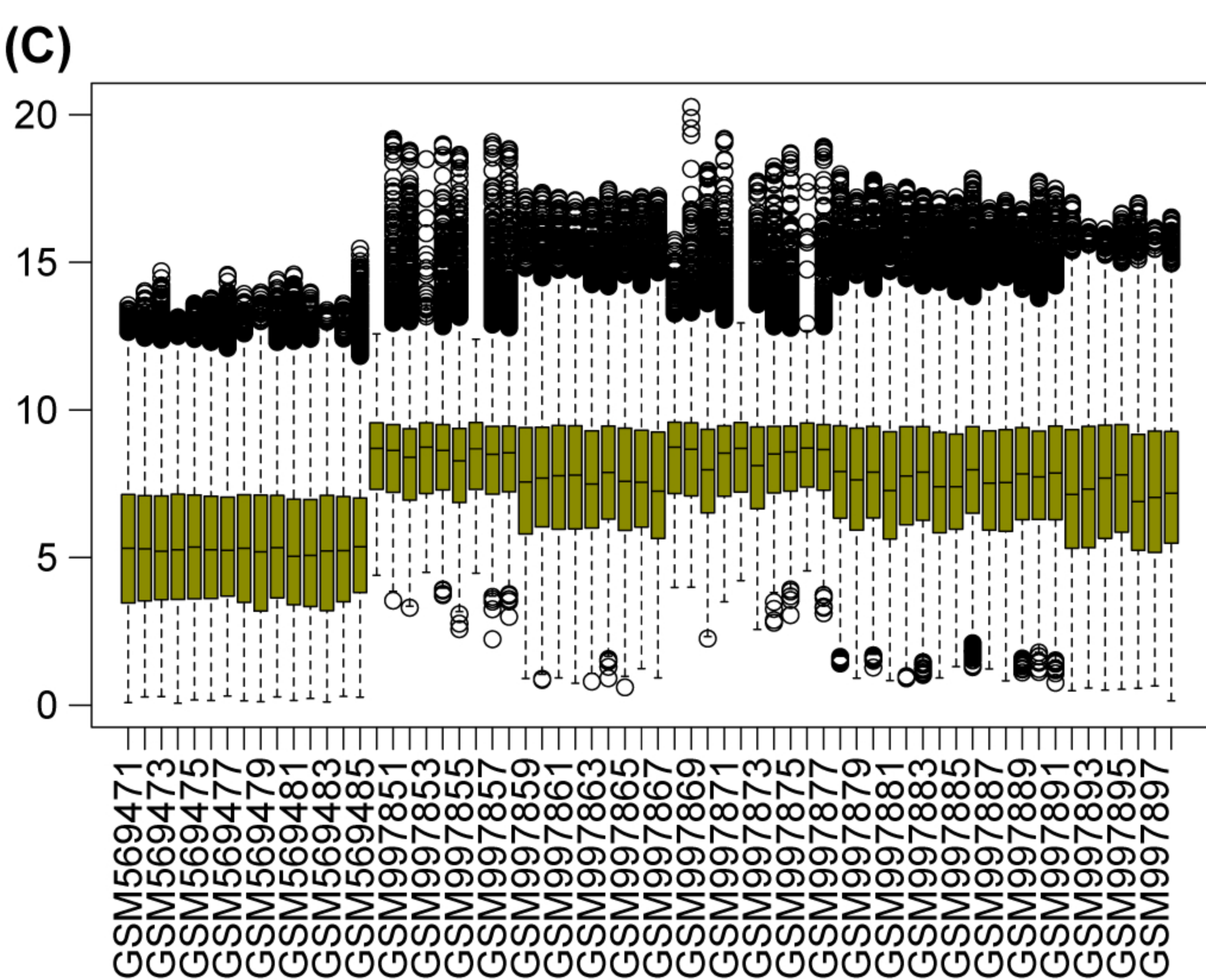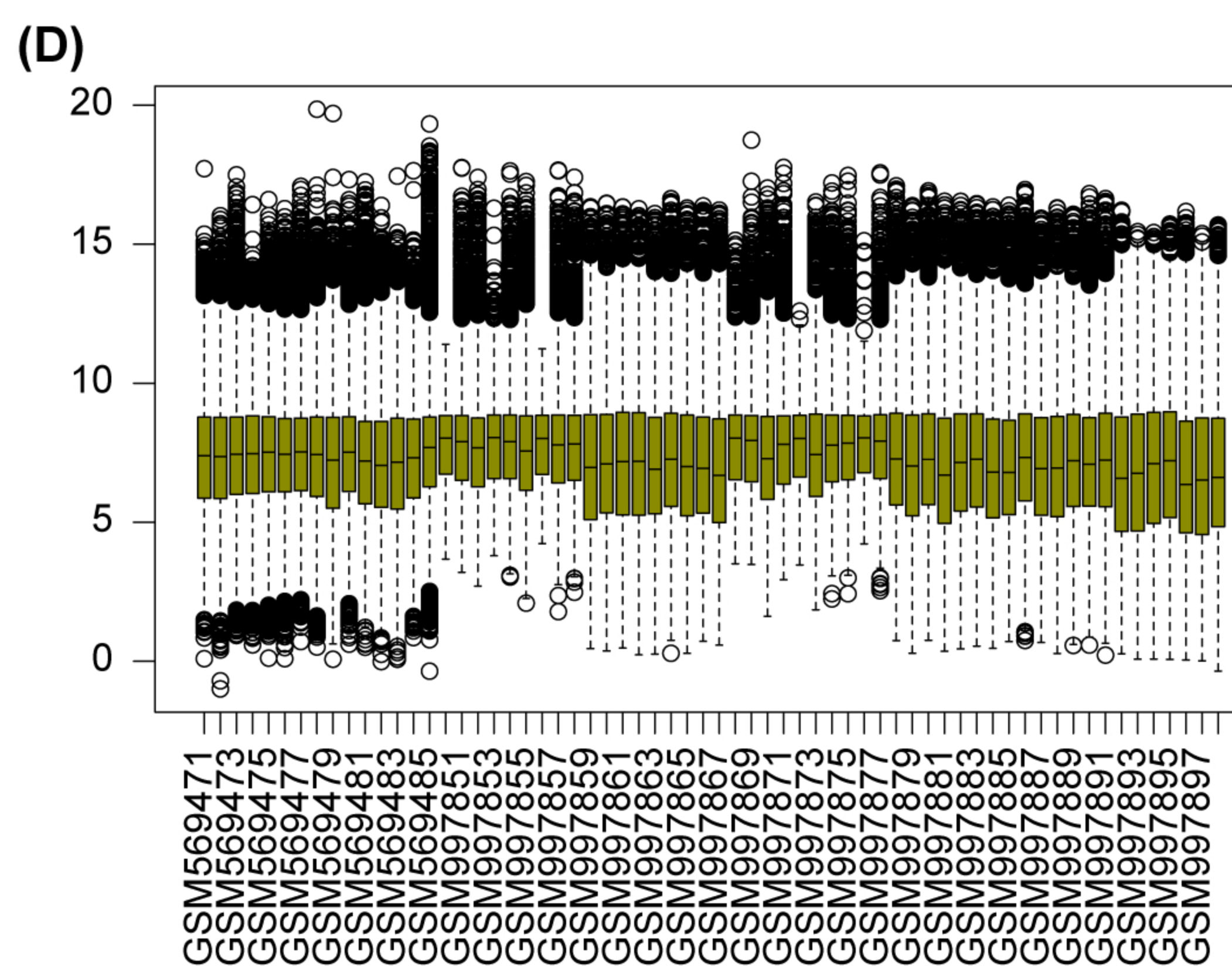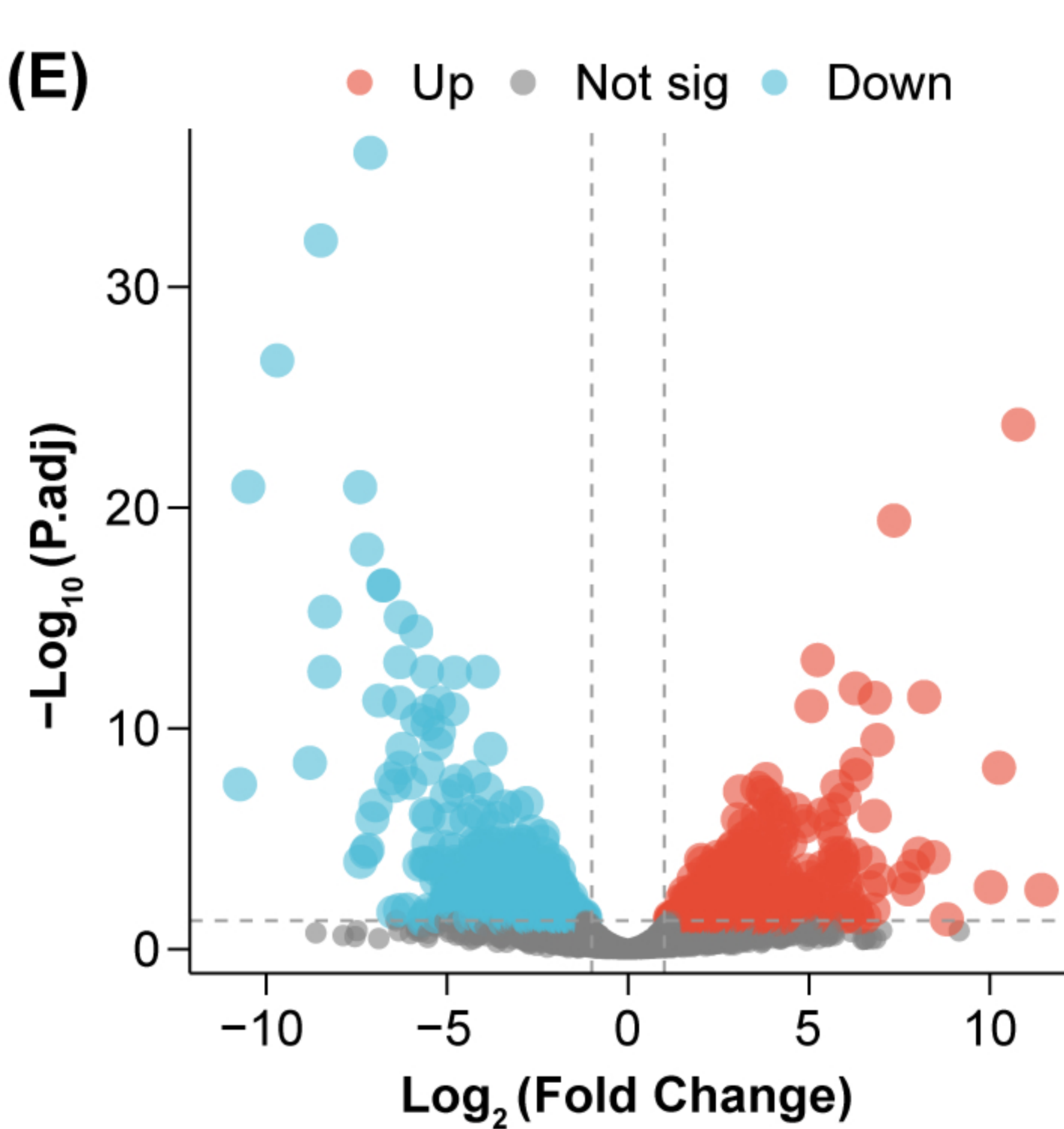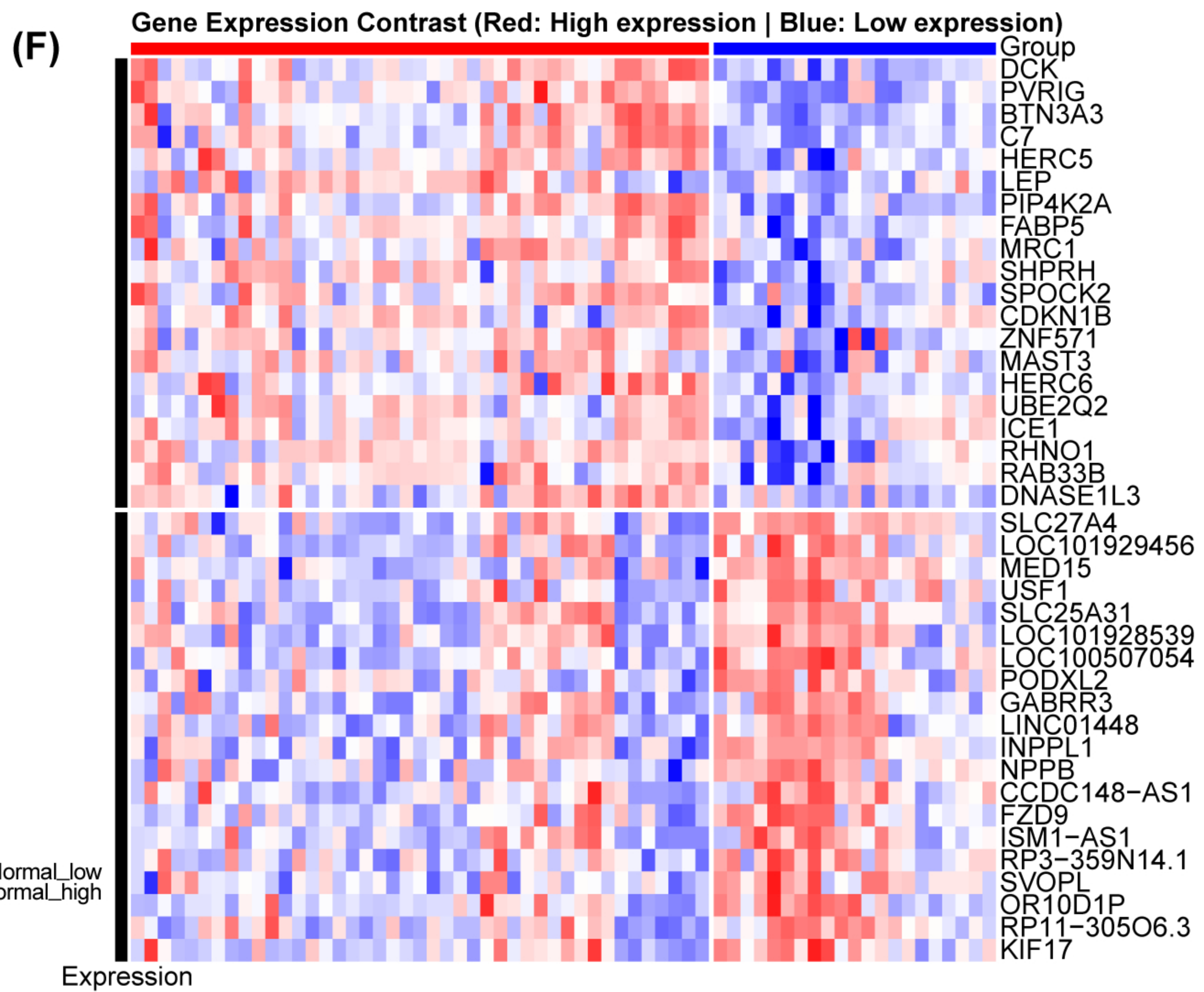

Supplement: Supplementary file 1 [file ijms-27-03365-s001.zip › Supplementary Figures/FigureS1.pdf]

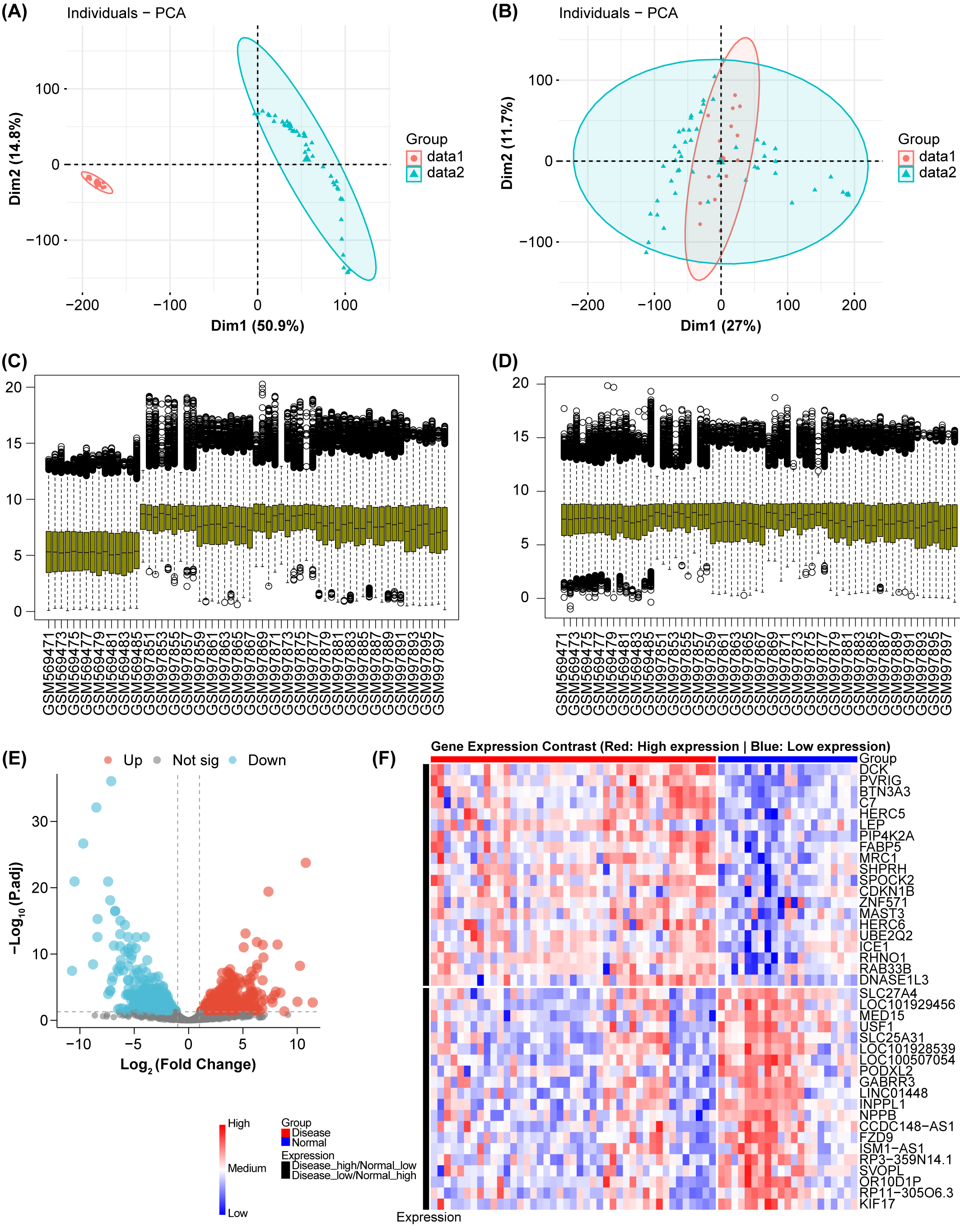

Supplement: Supplementary file 1 [file ijms-27-03365-s001.zip › Supplementary Figures/FigureS1.tif]

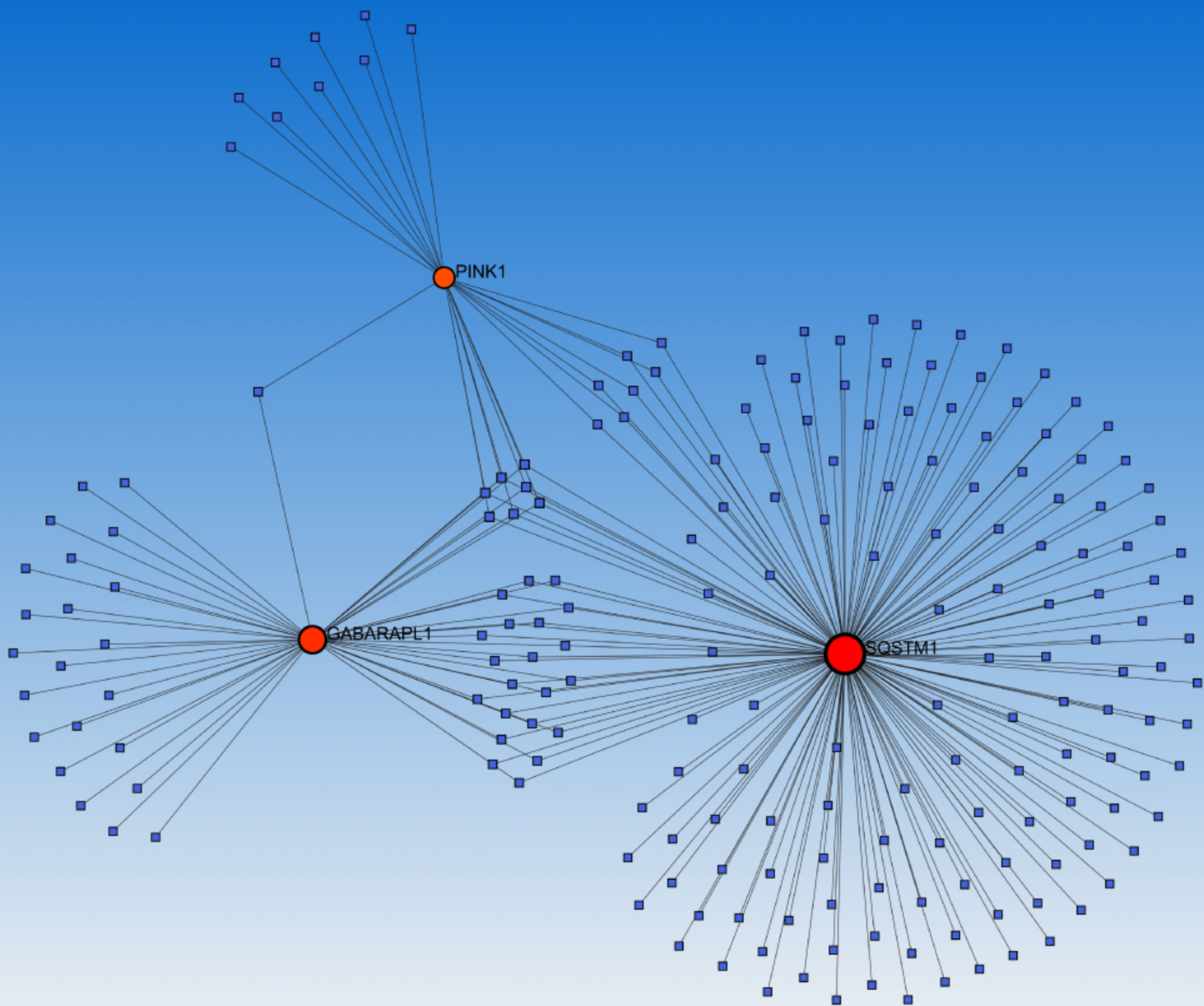

Supplement: Supplementary file 1 [file ijms-27-03365-s001.zip › Supplementary Figures/FigureS6.pdf]
